# Dynamical Behavior of Pure Spin Current in Organic Materials

**DOI:** 10.1002/advs.202207506

**Published:** 2023-03-30

**Authors:** Naihang Zheng, Haoliang Liu, Yu‐Jia Zeng

**Affiliations:** ^1^ Key Laboratory of Optoelectronic Devices and Systems of Ministry of Education and Guangdong Province College of Physics and Optoelectronic Engineering Shenzhen University Shenzhen 518060 P. R. China; ^2^ Guangdong Provincial Key Laboratory of Semiconductor Optoelectronic Materials and Intelligent Photonic Systems School of Science Harbin Institute of Technology in Shenzhen 518055 Shenzhen P. R. China

**Keywords:** magnon, organic spintronics, pure spin currents, spin pumping, spin Seebeck effect

## Abstract

Growing concentration on the novel information processing technology and low‐cost, flexible materials make the spintronics and organic materials appealing for the future interdisciplinary investigations. Organic spintronics, in this context, has arisen and witnessed great advances during the past two decades owing to the continuous innovative exploitation of the charge‐contained spin polarized current. Albeit with such inspiring facts, charge‐absent spin angular momentum flow, namely pure spin currents (PSCs) are less probed in organic functional solids. In this review, the past exploring journey of PSC phenomenon in organic materials are retrospected, including non‐magnetic semiconductors and molecular magnets. Starting with the basic concepts and the generation mechanism for PSC, the representative experimental observations of PSC in the organic‐based networks are subsequently demonstrated and summarized, by accompanying explicit discussion over the propagating mechanism of net spin itself in the organic media. Finally, future perspectives on PSC in organic materials are illustrated mainly from the material point of view, including single molecule magnets, complexes for the organic ligands framework as well as the lanthanide metal complexes, organic radicals, and the emerging 2D organic magnets.

## Graphical abstract

In this paper, recent experimental progress of the generation, the propagation and the detection of the pure spin currents in organic materials including the organic semiconductors and the organic magnets are explicitly reviewed and discussed revolving around the spin pumping and spin Seebeck techniques, which provides further perspectives to organic spintronics from molecular magnetization dynamics point of view.

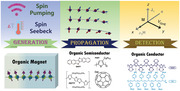

## 1 Introduction

The fact that electrons can conduct charge laid the basic foundation for the electronics industry during the past century. Since the 1920s, another inherent degree of freedom for electron named spin was discovered, which ultimately fuels a profound influence on the modern industrial data processing and storing technology.^[^ ^1^ ^]^ Julliere's finding of tunneling magnetoresistance (TMR) in the 1970's and the 1980's successful observation of giant magnetoresistance (GMR) phenomenon further demonstrated the spin's market potential beyond labs, for prominent instances of magnetic sensors and hard disk drives in computing, automated and biomedical equipment.^[^ ^2^, ^3^, ^4^ ^]^ Almost in the contemporaneous period, another novel class of conductive materials, named organic semiconductors (OSCs), was miraculously invented under the co‐work of Hideki Shirakawa, Alan MacDiarmid, and Alan Heeger.^[^ ^5^ ^]^ Such material class likewise wakened a tremendous revolution on the electronics industry within perspectives of large area solution‐processibility, chemical tailoring and the wearable device fabrication.^[^ ^6^, ^7^ ^]^ Note that the light element composition of organic semiconductors, longer spin lifetime in range of 10^−7^–10^−5^ s is expected in these molecules compared to the traditional metals and III–V group inorganic semiconductors.^[^ ^8^ ^]^ However, the successful practical conjunction between these two Nobel prize disciplines was vacant for a long time until 2004, Xiong successfully achieved organic GMR signal in vertical spin valves containing Alq3 (one representative commercial OSC active layer molecule in the organic light emitting diode).^[^ ^9^ ^]^ Such milestone result not only pointed out the available path to utilize OSC materials in the spin functional devices, more importantly, triggers a new discipline, as defined “Organic Spintronics”.^[^ ^10^ ^]^ Since then, growing members of OSC molecules were adopted in the spintronics devices, leading to continuous fancy spin discoveries apart from simple MR response.^[^ ^11^, ^12^, ^13^ ^]^

During the first decade before 2010, research scope of organic spintronics mainly concentrates on seeking the valid organic semiconducting transport media in organic spin valve (OSV) device, which also encompasses improving spin injection efficiency and developing physical models.^[^ ^14^ ^]^ Besides, multi‐functionality operation, such as spin light emitting diode (LED) and nonvolatile spin‐based electrical output were also achieved in coordination with single OSV unit.^[^ ^15^, ^16^ ^]^ In the meantime, questions such as the origin of negative MR, finite spin diffusion length and obscure relaxation mechanism left a huge space for further spin exploration even till now.^[^ ^17^ ^]^ Within the next decade until 2020, spintronics field experienced an huge revolution from both physics and material points of views. Spin orbital coupling, which is a pivotal driving force of the conversion between spin and charge, receives surging concern thus finally develops into a fascinating direction named spin orbitronics.^[^ ^18^ ^]^ Such discipline endows new vitality to both designing the spin‐based materials across multi‐dimensional and further tuning magnetism by spin torques under the electrical stimuli.^[^ ^19^, ^20^, ^21^, ^22^ ^]^ As for the molecule‐based organic spintronics branch, impressive advances have also been made during the same stage. In‐depth investigations of charge‐accompanied spin polarized current were still undertaken, covering details from the bulk to the interface, plus further enrichment on both the functionality and material warehouse.^[^ ^23^, ^24^ ^]^ On the other hand, generation, propagation and detection of pure spin current (PSC) in organic compounds were also probed and discussed recently as inspired by the inorganic counterpart.^[^ ^25^, ^26^, ^27^ ^]^ Encouraging results were gained, from another angle, further answered traditional bewildering questions in the past organic spintronics research as illustrated above. More importantly, PSC‐themed exploration can promote the spintronics better integrate with organic compounds regardless of limitations from material conductivity, device size and crystalline lattice match.^[^ ^28^ ^]^ Molecular magnetism which intensively links synthesis chemistry and magnetic physics holds great potential in quantum computation since the 1980s.^[^ ^29^ ^]^ Material carrier namely molecular magnet can either minimize into the single molecule size or expand to multi‐dimensional by aid of the chemical ingredient engineering or even van der Waals force.^[^ ^30^, ^31^, ^32^ ^]^ Related application in the spin polarized current devices has also been attempted successfully by serving as both the polarized electrodes and the transport medium.^[^ ^33^, ^34^, ^35^ ^]^ Nevertheless, the rich spin physics behind the molecular magnets is far‐less unveiled in functional devices beyond spin‐valve‐like devices, by advancing form such as magneto–opto coupling and terahertz technique.^[^ ^36^, ^37^ ^]^ Migrating eyesight to the key of the current chip industry, continuous doubt on Moore's law and attempts as well for breaking von Neumann bottleneck prompt the researchers seeking technical alternatives beyond the traditional CMOS technology. Spintronics arouse surging and extensive interests as a critical academic pillar GMR‐based readheads to the present hot‐discussing magnetic random‐access memories (MRAM).^[^ ^38^, ^39^ ^]^ The latter spin information manipulating platform is more reliant on the dynamical magnetic order or torque activity, which can be modulated in either electrical stimuli or optical radiation.^[^ ^40^, ^41^ ^]^ In summary of above facts, PSC study displays great significance on affording both the theoretical guidance and the practical support for further prosperity of molecule‐based spintronics, as well as developing low energy‐cost and high computing speed devices.

The framework of this PSC‐themed review is organized as follows: We first elucidate the basic concept of pure spin current, by afterwards of physical generation regimes and detection methods. Then pioneering exploration examples of PSC in the organic materials were demonstrated, including semiconductors and molecular magnets. In the meantime, several key questions in organic spintronics were also discussed, such as the debate of the spin injection and transport and the charge influence on the spin transmission. Finally, recent novel PSC discoveries involving optical and chemical combination were displayed by ending of further prediction mainly from material perspectives including the single molecule magnets (SMMs), the recent hot‐discussing organic ligands framework as well as the lanthanide metal complexes, organic radicals and the emerging 2D organic magnets. We anticipate this review can inspire more judicious findings balancing both material project and spin physics in not only organics electronics, but also other frontier research fields.

## 2 Basic Concepts of Pure Spin Currents

### 2.1 Categories of the Spin Currents

**Figure 1:**
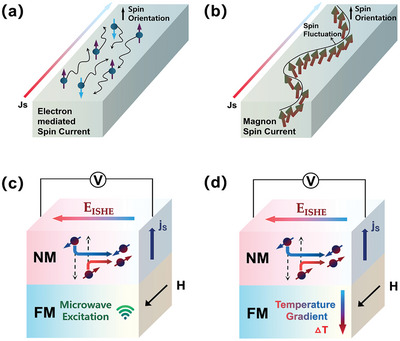
Category and generation mechanism of the spin currents. a) Electron‐mediated spin currents; b) magnon spin current; c) diagram for spin‐pumping; d) diagram for spin Seebeck effect.

Definition of the spin currents generally refers to the electron‐mediated spin currents, which contains both the electron and the spin information processing carriers as depicted in **Figure** **1**a. More accurately speaking, the electron‐mediated spin currents contain both the spin polarized currents and the electron‐mediated pure spin currents. The former spin polarized current concept indicates both spin‐up and spin‐down degrees are mediated by the conducting electrons and diffuse in the same direction as the electron carriers move. Such phenomenon generally happens in the electrical biased magnetic system, in other words, represent the spin polarized injection. The well‐known GMR or TMR effect are established on the spin polarized currents. As for the pure spin currents, the nonmagnetic conductive materials can serve as the generation resource by aid of the spin Hall effect.^[^ ^42^ ^]^ Under such circumstance, the equilibrium spin‐up and spin‐down carriers drifts oppositely, thus resulting in net zero charge flow while inducing pure spin current perpendicular to the polarized direction. One facile discrimination between these two conduction electron spin currents is the existence of charge accumulation. Strictly, conductive charge carriers are not the prerequisite for pure spin current generation. Torques in the magnetic insulators can be collectively excited and finally forms spin waves, even by absence of conductive carriers. Such angular momentum flow can also be named as magnon (quanta of spin wave) spin current^[^ ^43^, ^44^ ^]^ as displayed in Figure 1b. Early probing history of such wave‐like spin current can be dated back to 1929 by Bloch, while the modern systematic investigation especially for detection aroused no longer than past 10 years. This emerging quasi‐particle current brings new perspectives to design information processing units below CMOS technology size as well as higher efficiency logic‐integrated circuits and wave‐based computing networks.^[^ ^45^ ^]^

Undeniably, coupling between spin and charge, or by another form namely spin polarized current, leads to a huge revolution in the electronics industry. However, the accompanied Joule heat can hardly be ignored accounting for the consequent energy consumption and the probable advanced device worn‐out deadline.^[^ ^46^ ^]^ Charge‐free pure spin current caters well to the increasing environmental‐friendly appealing, meanwhile, further promotes the spin‐tongued disciplines progressing from the traditional theoretical framework to the future advanced industrial application. Generation mechanism of PSC encompass a plenty of spin physics questions and also determines the attributes of spin angular momentum flow. Two representative mechanisms will be elaborated in the next section.

### 2.2 Mechanism for Pure Spin Current Generation

#### 2.2.1 Spin Pumping

Magnetization behavior in the ferroelectric materials is subject to the external electrical bias as early discovered in the 1940s.^[^ ^47^ ^]^ Similar magnetism tuning phenomenon can also be realized in various magnets by simply exchanging pure spin currents with the magnetic torques, thus finally drives magnetization precessing even without the external magnetic field assistance. Oppositely, spin pumping is the inverse process beginning from exciting magnetization precession to pure spin current emission, in most cases, driven by the ferromagnetic resonance (FMR) technique.^[^ ^48^ ^]^ The first report of the spin pumping can be traced back to 1970s by Silsbee when investigating the transmission‐electron‐spin‐resonance of Cu.^[^ ^49^ ^]^ Such dynamical spin injection method is applicable to generate either electron‐mediated or magnon‐based pure spin currents according to the massive experimental attempts.^[^ ^50^ ^]^ Complete spin‐pumping procedure encompass following three key steps: 1) Driving magnet's macroscopic magnetization into the resonant precessional states by simultaneously applying external static in‐plane magnetic field and continuous microwave radiation. 2) Pumping the excess unrelaxed angular momentum across the magnet/non‐magnet interface to achieve dynamical spin‐exchange injection. 3) Detecting the spin‐pumping induced pure spin currents by the resulting converted electrical or the optical signals. Step 1 is the nontrivial requisite for Step 2 smoothly undertaking, while Step 3 is a feedback of Step 2 examining whether spin current transmission overwhelming backflow. As depicted in Figure 1c, such “pumping” spin injection calls for the collaboration between bulks and interfaces, naturally involves a vast of physical and technical questions. Both electrically conductive (metallic or even semiconducting) magnets and insulating magnets can be exploited for generating pure spin currents during precessional excitation.^[^ ^51^, ^52^ ^]^ The former, such as metallic NiFe and semiconducting (Ga,Mn)As, generally results in electron‐mediated pure spin current. As for the magnetic insulator, taking instance of the most‐investigated Ytrium Iron Garnet (YIG), are widely used for nonlinear spin pumping which leads to magnon‐based pure spin current. Worthy of emphasizing, the stronger magnetization excitation under a sufficiently large frequency microwave is the primary consideration to acquire large spin current in the magnet/non‐magnet hybrid junction during the spin‐pumping operation, whose importance somewhat exceeds the specific option on the magnet. The neighboring non‐magnetic part plays a dual role of both transmitting pure spin current across the magnet/non‐magnet interface and detecting the spin pumping injection efficiency. The efficiency and the density of spin current pumped across the magnet/non‐magnet interface can be evaluated by the below equation:

(1) jspump=ℏ4πReg↑↓m×dmdt−Img↑↓dmdt

Herein, **j_s_^pump^** refers to the pumping‐induced spin current density, while ***g* ^↑↓^** stands for the spin mixing conductance, **m** is the magnetization orientation unit vector, and ℏ is the reduced Planck constant. The spin mixing conductance ***g* ^↑↓^** which quantitatively defines the available channels for transporting the pumped unrelaxed angular momentum flow, is the core of such formula and positively proportional to the spin injection efficiency. According to the past investigations, bi‐layer structures consisting of the low magnetic damping, the high spin polarization magnets and superior conductivity, large SOC non‐magnets are more preferred for obtaining large ***g* ^↑↓^** value. Selection for the magnetic part has been illustrated just before thus following discussion will be transferred to the non‐magnetic counterpart. Preliminary attempts for the spin pumping experiments were mainly launched among the metallic bi‐layer structures whose non‐magnetic part is the heavy 3D transition metal, such as Au or Pt with ***g* ^↑↓^** magnitude dwelling around the order of 10^19^ m^−2^.^[^ ^53^ ^]^ Pure spin current detection, which is the final circle of complete spin pumping experiment, can be achieved either spectroscopically or electrically. In contrast to the single excited magnet during the spectroscopic detection, the damping enhancement of magnet/non‐magnet system is the direct proof for the efficient pure spin current generation and injection.^[^ ^54^, ^55^ ^]^ As for the electrical detecting method, the inverse spin Hall effect (ISHE) driven, measurable DC voltage is the core signal which can be simultaneously applied for estimating the spin‐to‐charge conversion.^[^ ^56^, ^57^ ^]^ Compared to the spectroscopic PSC detecting method, electrical protocol can afford better quantitative platform to evaluate the spin–orbit interaction, thus is predominantly considered in examining the spin pumping efficiency. Merits of feasible room temperature operation and no rigid demand for conductivity mismatch make spin‐pumping increasingly popular on investigating dynamics of pure spin itself and enhancing the spin injection efficiency. On the other hand, combination of “Spin‐pumping Generation”and “ISHE Detection” has become an essential even the first‐class studying route for probing the pure spin current issue. In the early studies, organic materials were considered as the unsuitable candidates for the spin‐pumping experiments due to its hopping‐dominated low carrier mobility and inherent weak spin–orbit coupling. These characteristics, generally speaking, is unfavorable to obtain the decent spin‐mixing conductance and ISHE‐induced electromotive DC signal. In addition, bulk films processed from organic molecules are mostly fragile and even unstable, hence raise huge challenges for spin‐pumping projects. In spite of these shortcomings, the long spin lifetime and the low‐cost, high‐flexibility, structural‐dependent conducting performance make the chemical‐versatile organic materials still appealing for the further spintronics research, definitely including pumping‐induced pure spin current issue. Recently, the probing vacancy for the organics‐based spin pumping has been filled up thanks to the unremitting efforts of both spintronics and material community.^[^ ^58^ ^]^ Either magnetization precession resource for pumping spins or non‐magnet spin‐to‐charge terminal has been illustrated possible in organic networks.

#### 2.2.2 Spin Seebeck

Seebeck effect has witnessed a brilliant exploring journey over the past two centuries since T. J. Seebeck discovered an electrical voltage can be generated in conductors when they are imposed external temperature gradient.^[^ ^59^ ^]^ The spin Seebeck effect, literally, the spin analogy of the Seebeck effect, converts the imposed temperature gradient to a non‐equilibrium spin voltage (current). In 2008, Uchida first observed the spin Seebeck effect (SSE) in the NiFe/Pt bilayer structure where the spin voltage thermally generated in the NiFe film was finally converted to the electrical voltage driven by the ISHE effect.^[^ ^60^ ^]^ Such innovative discovery creates a facile route for studying the SSE phenomenon, that is exploiting ferromagnetic metal as the thermally‐induced spin resource, in combination with the non‐magnetic heavy metal which is responsible for the ISHE‐induced electromotive spin detection. Note that in Uchida's SSE pioneering attempts, authors tentatively attributed the thermodynamics‐generated PSC to the thermally spin accumulation within the NiFe layer, borrowing lessons from normal metal's Seebeck effect. In other words, the spin‐up and spin‐down channels possessing different thermopower is the core driving force of SSE‐generated spin currents. Whereas, two intriguing observations seemingly contradict such viewpoint: the first is the discrepancy that Uchida's initial SSE was a non‐local effect, contrary to the local character in traditional Seebeck effect observed in normal metals. The second also the most extrusive, is the sustaining distance for the SSE even reached millimeter size, far exceeding the spin relaxation length of metals. Plausible explanation for the new born SSE phenomenon is in lack until Xiao proposed pumping‐like thermal‐activated interface spin exchange in 2010. In his discovery, successful SSE observation did not rely on the conductive motional carriers, instead, can be achieved in magnetic semiconductor (GaMnAs) even insulator (YIG) based configuration, as concurrently supported by Jaworski and Uchida's experimental results.^[^ ^61^, ^62^ ^]^ These facts further indicate the spin Seebeck effect is relevant to the magnetization dynamics, and more importantly, can be universally observed in the magnet family. In this context, a new discipline called the spin caloritronics aroused, which concentrate on the interplay between microscopic spin degree and macroscopic heat management.^[^ ^63^, ^64^ ^]^

Experimentally, transverse and longitudinal configurations are the two main architectures for characterizing the spin Seebeck effect.^[^ ^65^ ^]^ Temperature gradient applied perpendicular to the pure spin current flowing direction corresponds to the transverse configuration, while the longitudinal configuration holds the parallel alignment between the temperature bias and the resulting pure spin current. The former although is the very first experimental demonstration and more tolerant on the magnet conductivity, accompanied complex manipulation over temperature distribution and severe substrate dependence make it unideal to evaluate SSE origin accurately. Thereby, herein we will only discuss the longitudinal spin Seebeck effect like most recent experiments suggests.^[^ ^66^ ^]^ As depicted in Figure 1d, temperature gradient was applied parallel to the spin current emitting direction thus enables a direct and facile way to measure the SSE. Neighboring non‐magnet section still plays the role for the thermal pumping induced PSC detection, manifested by the inverse spin Hall voltage. Punctually speaking, the longitudinal spin Seebeck effect (LSSE) face the limit on the magnet selection, that is only the insulating magnet can be employed accounting for the disturbance of anomalous Nernst effect in conductors. Nevertheless, such constraint offers a good opportunity for utilizing low‐damping magnetic insulators and to some degree, better promote the combination between magnonics and thermonics. Groundbreaking attempts involving the organic materials have been also reported just recently.

The stunning success of organic giant magnetoresistance two decades ago illustrated the nontrivial role of OSCs in organic spintronics.^[^ ^24^ ^]^ However, direct pumping spins solely from the organic semiconductors by whether microwave excitation or temperature gradient has not been achieved hence calls for the additive magnetic resources. Magnetic metals, as the material cornerstone of spintronics, have already fruited two mature spintronics commercial products: MRAM and hard disk drives as demonstrated above.^[^ ^3^ ^]^ Stable non‐equilibrium spin resources and wide compatibility with substrate either hard or flexible make the magnetic metals still appealing for PSC especially generation study. Whereas, generating PSC directly from the ferromagnetic metal spin systems to OSCs face evident challenges not only due to the ferromagnetic metallic surface prone to oxidation, interface conductivity mismatch and possible spin backflow should also be considered. By contrast, inferior conductivity of magnetic semiconductors and insulators seemingly can afford better spin transparency when contact directly with semiconducting organics. Featured by the exceptionally long spin‐coherence and gate tunable conductivity, the ferromagnetic semiconductor has motivated a collection of fascinating spin discoveries involving coupling between spin and electrical current, heat, and light.^[^ ^67^ ^]^ Representative material GaMnAs family has been demonstrated valid for both thermally driven or microwave excited spin angular momentum flow generation.^[^ ^68^ ^]^ Note that the low Curie temperature due to the dilute magnetic behavior, direct ferromagnetic semiconductor conjunction with OSCs is hard to be undertaken under room temperature. As for the magnetic insulators which brings a huge revolution in magnonics,^[^ ^45^ ^]^ the insulating garnet ferrite with low damping parameter and good stability is an ideal material candidate to generate PSC especially magnon flow even in direct contact with OSCs. While the expensive fabrication cost and the rigid film growth conditions of the garnet ferrite hinders the expecting large‐area, flexible spin‐based units integration. As seen, pros and cons for using magnetic metals, semiconductors or insulators to generate PSC into organic semiconductors are explicitly discussed. Both the advantages and the drawbacks can offer good reference and opportunities to the further exploration of PSC in OSCs from different angles. Moreover, organic magnets though compromise in the conductivity by contrast with OSCs, can also exhibit promising perspectives in PSC generation. Following sections will give a detailed elaboration of representative breakthroughs for the dynamical behavior of PSC in organics.

## 3 Spin Pumping‐Induced Pure Spin Current in Organics

### 3.1 Pure Spin Current Detection in Organic Semiconductor

**Figure 2:**
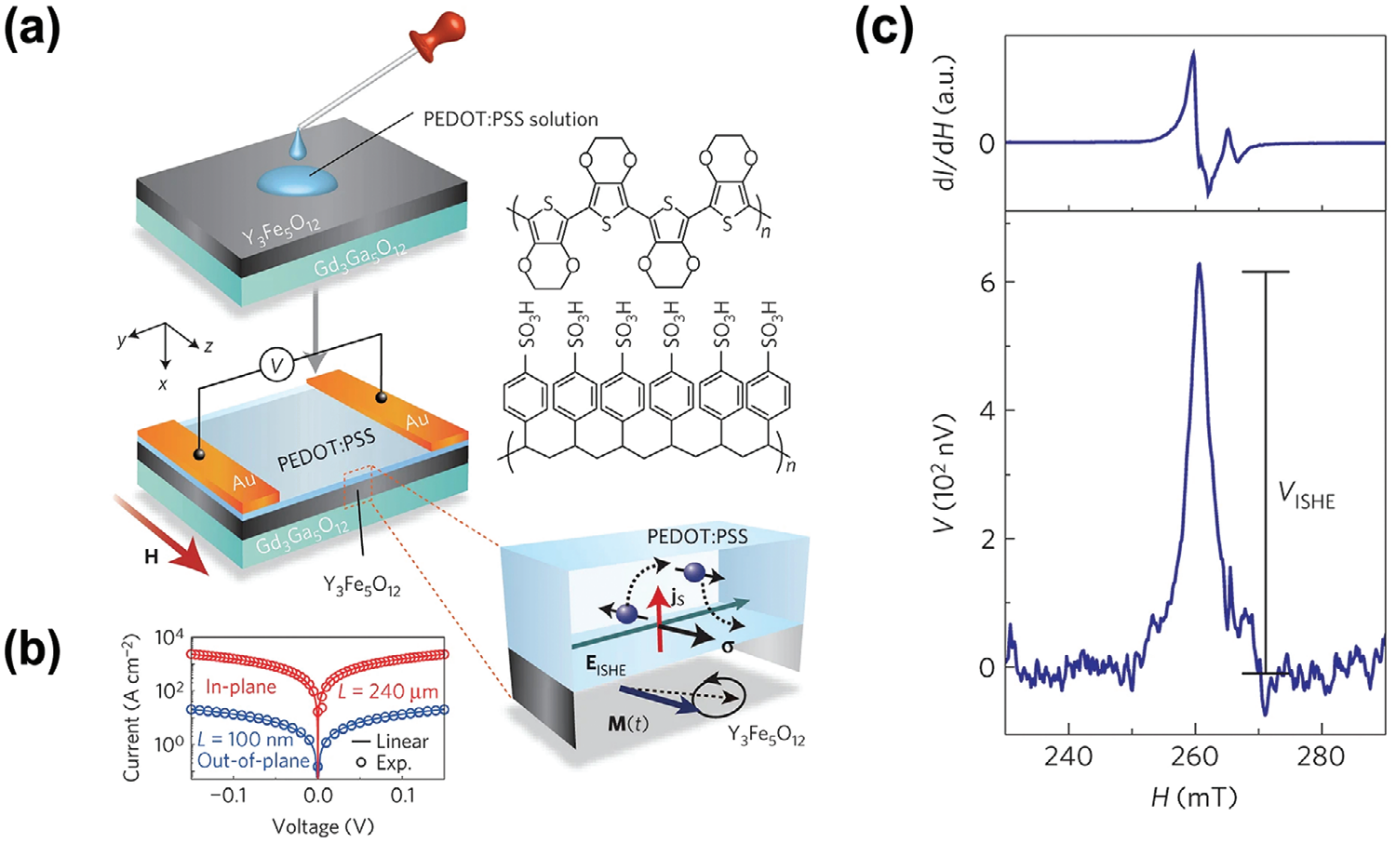
The first discovery of spin‐to‐charge conversion in solution‐processed conducting polymer PEDOT: PSS. a) Schematic display for the YIG/PEDOT: PSS bilayer and the polymer structure information. b) Current‐voltage characterization results of the solution‐processed PEDOT: PSS layer. c) Field dependence of the FMR signal d*I*/d*H* and the electromotive voltage response for the PEDOT: PSS/YIG at 20 mW microwave excitation. Reproduced with permission.^[^ ^69^ ^]^ Copyright 2013, Nature Publishing Group.

The first successful organic pure spin current was reported in 2013, that Ando creatively selected the conductive poly(3,4‐ethylenedioxythiophene): poly(4‐styrenesulphonate) (PEDOT: PSS)polymer as the detecting terminal for pure spin current generated from the single‐crystal ferrimagnetic insulator Ye_3_Fe_5_O_12_ (YIG) film via spin‐pumping method.^[^ ^69^ ^]^ Device configuration and polymeric structural information are demonstrated in **Figure** **2**a. PEDOT:PSS has a high in‐plane conductivity up to 10^3^ S cm^−1^, which is beneficial to undertake the high‐sensitivity, low‐noise ISHE detective measurement. Reasons for the specific option of YIG instead of the traditional ferromagnetic metals owes to three main respects: 1) the ultra‐high resistivity up to 10^12^ Ω for the YIG film offers a good platform to analyze the electromotive DC voltage signal transformed in the detective conducting polymeric film void of spin pumping resource contribution. 2) Insulating nature of ferrimagnetic YIG film can erase the disturbance of parasitic voltage generated by anomalous Hall effect (AHE), spin backflow or anisotropic magnetoresistance in traditional ferromagnetic metals, such as NiFe. 3) Considering the adhesive conductive polymeric thin film is completed by spin‐coating artefact, utilizing oxide‐based YIG film instead of traditional ferromagnetic metals can effectively hinder the magnet contamination. To ensure the spin‐pumping experiments undergo smoothly, authors firstly examined the electrical conductivity of such Polymer/YIG based heterostructures in both in‐plane and out‐of‐plane directions. Resulting large anisotropy current‐voltage characteristics are shown in Figure 2b, which is a good indicative of the uniform alignment of the polymer thin film aggregation. Subsequently, authors exerted a GHz‐level frequency microwave in concert with a static magnetic field along the film plane to induce ferromagnetic spin–wave resonance in the YIG film. Under the FMR resonance conditions, pure spin currents are injected into the conductive polymer film from the precessing YIG magnet, and ultimately converted to a measurable electrical voltage across the two Au electrodes. Figure 2c displayed the microwave absorption induced FMR signal and the converted DC voltage response in the PEDOT: PSS layer under room temperature. As depicted, the resulting symmetric *V*_ISHE_ curve shares the identical (*H*_FMR_ = 260 mT) resonance field with FMR signal, which seemingly indicates the measured DC voltage across the polymer thin film arisen from the ISHE effect. In fact, various interfering spin phenomenon, such as the organic magnetoresistance and the spin thermoelectric effect, should be treated seriously before asserting the macroscopic electromotive signal is solely driven by the desired ISHE effect. Afterward, authors performed a collection of parallel tests, like modulating the relative angle between the external static magnetic field and the films stacking plane, or alternating the microwave frequency and finally testified the observed voltage was irrelevant to the electromagnetic and spin thermoelectric effect. After proving the experimental observations in ferrimagnetic/conducting polymer hybrid structure exhibited the spin‐pumping characteristics in respects of the angle, frequency, power dependence, and subsequently evaluate the spin‐to‐charge conversion efficiency by contrast with reference YIG/Pt junction, an representative classical pure spin current investigation configuration.^[^ ^70^ ^]^ Indeed, the considerable spin–orbit coupling strength and the resulting large spin Hall angle of the Pt layer resulted in stronger voltage signal, which is two orders larger than that observed in the solution‐processed polymer sample. Nonetheless, relative value for the spin‐to‐charge conversion efficiency in conductive PEDOT: PSS polymer is miraculously comparable to that in the heavy atom Pt film after simulation. This can be explained by the intrinsic conductivity anisotropy of PEDOT: PSS film, where pumping‐induced pure spin current flows along the low‐mobility out‐of‐plane direction and conversely, transformed charge flow accumulates and diffuses into the high‐mobility direction. This work sets a good beginning and guidance for investigating pure spin currents in light, flexible, conductive organic molecules, especially *π*‐conjugated polymers. In addition, another novel physical issue arises, namely “Spin Hall effect in the hopping organics”, which bear great significance for better understanding of spin–orbit interactions in the hopping‐dominated organic semiconducting networks. Subsequently, the other widely studied OSC members were also attempted for investigating spin‐to‐charge phenomenon for instances of Alq3,^[^ ^71^ ^]^ pentacene,^[^ ^72^ ^]^ TIPS‐pentacene,^[^ ^73^ ^]^ C_60_,^[^ ^74^ ^]^ and polyaniline.^[^ ^75^ ^]^ Noteworthy, in‐depth studies concerning influence of the charge carrier mobility, molecular structural conformation on spin angular momentum dynamics were also probed which will be thoroughly discussed later in below sections.

### 3.2 Pure Spin Current Generation in Organic Magnet

**Figure 3:**
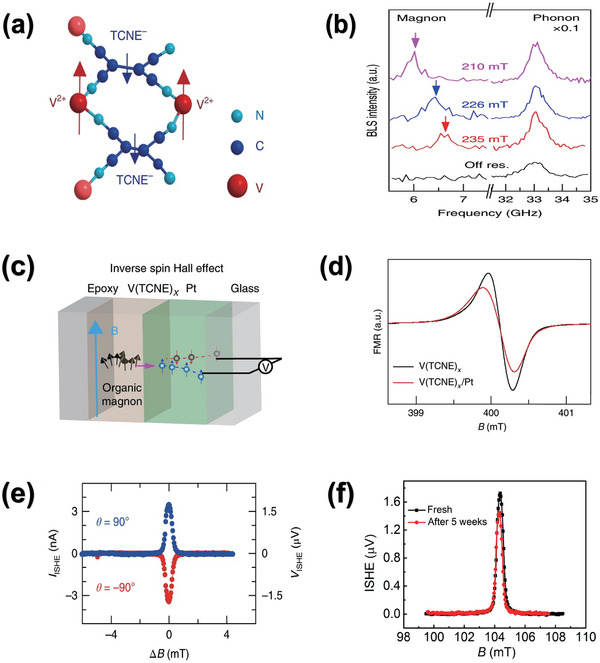
V(TCNE)x‐based organic magnon research. a) The magnetic structure of V(TCNE)x. The V^2+^ and (TCNE)^−^ spins couples by means of antiferromagnetic coupling. b) The BLS spectroscopic data measured at various magnetic fields and matched MW frequencies. c) Schematic diagram of ISHE‐induced magnon current electrical transformation process across the V(TCNE)/Pt interface. d) FMR spectra contrast between V(TCNE)x/Glass and V(TCNE)x/Glass nano‐structures. e) Converted *I*_ISHE_ signal under pumping resonance field in mode of continuous MW excitation. f) Ambient air stability of V(TCNE)x/Glass stacking. Reproduced with permission.^[^ ^81^ ^]^ Copyright 2018, Nature Publishing Group.

Magnonics further development mainly involves three perspectives: methods for the magnon generation and detection, comprehension over the magnon propagating regime and the enrichment of magnonics material warehouse.^[^ ^45^ ^]^ Among these three directions, explorations from material point of view are rather significant since they offer the critical guidance and support to the previous two directions. Over the past decade, low magnetic damping ferrimagnetic insulator YIG owing to the validity for emitting both the spin pumping‐driven coherent magnon current and SSE‐induced non‐coherent one, has been extensively exploited for spin wave studies.^[^ ^76^ ^]^ However, the most YIG film calls for rigid growing conditions, such as the liquid phase epitaxially‐formed single crystal under the high temperature. Moreover, obtaining high quality YIG thin film also demands supreme lattice match between growing substrate, which is generally constrained within GGG type. By contrast, organic‐based magnets are good alternative candidates because of the wider compatibility with substrate even the flexible ones. Furthermore, the intrinsic weaker spin–orbit coupling strength and the absent electron‐lattice scattering in organic magnets also favor the Gilbert damping reduction. V(TCNE)x, one representative organic magnet compound with good fabrication flexibility,^[^ ^77^ ^]^ has been reported available on constructing all organic spin valve.^[^ ^78^, ^79^, ^80^ ^]^ While the resulting limited MR value is deficient to showcase the spintronics potential of such molecular magnet. Migrating concentration toward its underlying dynamical magnetization phenomenon is a wise strategy, such as the PSC project. Under such context, Liu creatively achieved successful coherent magnon generation, transport and detection in V(TCNE)x based films and devices, by assistance of ferromagnetic resonance (FMR), Brillouin light scattering (BLS), and spin‐pumping techniques in 2018.^[^ ^81^ ^]^ The magnetic structure for such organic magnet was depicted in **Figure** **3**a, as seen, V^II^ ions and (TCNE)^−^ anions are coupled antiferromagnetically and results into ≈1 µB level magnetic moment. Growing artefact for the V(TCNE)x herein is selected into CVD method by reaction of V(CO)_6_ and TCNE on the already lithographically Cu‐patterned glass substrate. Afterwards, the magnetic nature of such organic magnet bulk film was thoroughly investigated including Curie temperature, saturation magnetization, and dynamical magnetic parameter. Noteworthy, low damping value around 3.2 × 10^−4^ was acquired, which is comparable to the inorganic hard YIG film. Before undertaking the routine FMR‐ISHE coalescent PSC investigation, Brillouin light scattering (BLS) spectroscopy was performed on the already glass‐grown sample to identify that magnons can authentically be excited in the V(TCNE)*_x_* film under FMR conditions. Related spectroscopic data at various magnetic fields (see Figure 3b) exhibited two peaks at both high and low GHz frequencies, thus indicated the BLS spectrum under resonance consists of both high and low energetic bands. The high energy around 33 GHz band corresponded to the light scattering due to the phonons in the glass substrate, which is insensitive to magnetic field alternation. Conversely, the low energy band below 7 GHz shifts with the field from 210 to 235 mT, as an indicative of magnon generation in the magnetic V(TCNE)x film during light scattering. MW frequency off control experiment was performed subsequently, and no magnon BLS band appeared thereby further illustrated the thermal magnon from the V(TCNE)x film contributed no larger than the noise level. These facts, together with the following examination about magnetic field dependence of BLS magnon frequency offer a solid proof to the authentic magnon generation in the V(TCNE)x film. Furthermore, magnon lifetime according to the linewidth obtained under low frequency region was also estimated in ns level. With these proofs, coherent magnon spin current emission and detection were finally performed in V(TCNE)x/Pt/Glass hybrid junction under both the pulsed and the continuous MW excitation as diagrammed in Figure 3c. V(TCNE)x/Glass bi‐layer was also prepared for the damping parameter contrast during the FMR measurement. In Figure 3d, the former Pt contained nanostructure possess larger damping value because of the enhanced magnon scattering loss at the organic/Pt interface. This further implied the magnon current through the V(TCNE)x film can transfer spin angular momentum more efficiently when surpassing Pt adjacent layer under the FMR conditions, also can lead to the larger spin mixing conductance. Figure 3e displayed the ISHE response acquired using the continuous MW excitation at the FMR states. Two representative significant features for FMR‐driven ISHE, such as narrow resonance peaks and angle‐dependent bias polarity reversal, could be both clearly observed in this ISHE signal picture. Furthermore, reference classical YIG/Pt bilayer was also fabricated and shared the identical measuring set‐ups with the aforementioned V(TCNE)x/Pt bilayer sample. Amazingly, the organic magnet sample exhibited the much narrower ISHE(B) response and no compromising strength compared with the well‐known YIG/Pt bilayer sample. Subsequently, pulsed ISHE experiments of the V(TCNE)_x_/Pt bilayer under the higher frequency (9.63 GHz) were also undertaken to not only monitor the time‐dependence of *I*_ISHE_, moreover, also to better rule out the potential influence of thermal contribution arising from the continuous and gradient heating artefacts. Fortunately, the resulting data further certificated the generation of pure spin currents were purely driven by the spin pumping supported by explicit formula calculation. Another unforgettable highlight for this work is the ambient stability of ISHE signal in V(TCNE)x/Pt structure which merely decayed nearly 20% after 5 weeks due to the additive epoxy protection (Figure 3f). This comprehensive and explicit investigations of magnon‐related phenomenon in organic magnet V(TCNE)_x_ created a novel direction in spintronics, namely organic magnonics, which holds a great bearing on designing the low‐cost, flexible magnon‐based logic devices.

### 3.3 Pure Spin Current Propagation in Organic Solids

**Figure 4:**
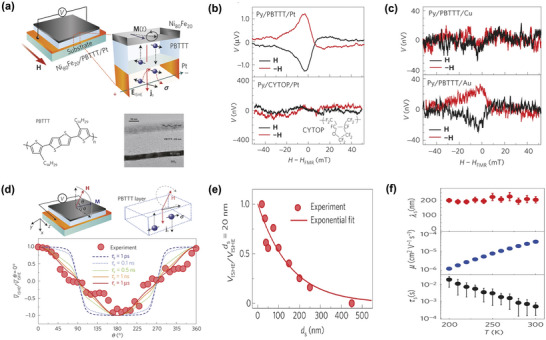
PBTTT‐based spin pumping experiment. a) Basic information of investigating PBTTT‐based spin pumping device including device geometry, polymer chemical structure, and device interface cross‐sectional transmission electron microscope image. b) Contrast of H dependence of the electromotive force *V*_ISHE_ measured for the Ni_80_Fe_20_ (10 nm)/PBTTT (40 nm)/Pt (7 nm) and Ni_80_Fe_20_ (10 nm)/CYTOP (60 nm)/Pt (7 nm) junctions. c) Contrast of H dependence of the electromotive force *V*_ISHE_ measured for the Ni_80_Fe_20_ (10 nm)/PBTTT (40 nm)/Cu (300 nm) and Ni_80_Fe_20_ (10 nm)/PBTTT (40 nm)/Au (40 nm). d) The concept of Hanle measurements and the out‐of‐plane magnetic field dependence of *V*_ISHE_ extracted by the fitting procedure from the voltage spectra measured for the Ni_80_Fe_20_ (10 nm)/PBTTT (60 nm)/Pt (7 nm) trilayer. e) PBTTT thickness dependence of the spin pumping trilayer *V*_ISHE_ magnitude. f) Temperature dependence of the spin diffusion length *λ*_s_, mobility *µ*, and spin lifetime *τ*_s_. Reproduced with permission.^[^ ^85^ ^]^ Copyright 2014, Nature Publishing Group.

In above neighboring two sections, we reviewed two pioneering PSC explorations in organics involving both the pure spin current generation and detection. Another question we should pay specific attention, that is the pure spin current propagation in the organic solids. Such issue holds great significance on comprehending and distinguishing dynamical behavior of spin angular momentum molecular networks. Differing from classical heavy metals and III–V groups semiconductors, charge carriers in organic semiconductors are localized 1/2 spin polarons which propagate dominantly by the hopping mechanism.^[^ ^82^ ^]^ Such transport nature is also responsible for the past debates on the spin diffusion performance and regime in such molecular media. Moreover, relaxation mechanism for spins within OSCs also face the similar situation like transport. Dominance of whether SOC or hyperfine interaction for relaxation is still a conflicting issue which is subject to molecule chemical structure and even morphology.^[^ ^83^ ^]^ Noteworthy, past related experimental routes of discriminating relaxation mechanism are mainly based on the vertical OSV device which afford finite spin diffusion length value and also encounter challenges from the conductance mismatch, interface quality and the successful room temperature operation.^[^ ^84^ ^]^ Besides, how charge carrier mobility influences spin dynamics in the hopping‐transport OSCs is a long‐standing debating issue, which obscures the precise selection of OSC spin transport media. These questions bewilder and urge the researchers to seek new insights and solutions to understand more accurately how spin propagates and decays in the hopping‐based molecular networks. PSC project caters well to such academic demand due to the separation of spin and charge during transport study as explicitly demonstrated above. After successfully pumping magnons from the insulating YIG film into the solution‐processed conducting and doped PEDOT: PSS polymer, Sirringhaus and colleagues subsequently probed the capacity of inherent polarons in undoped semiconducting polymer for carrying spin current as well as the relaxation mechanism yet by the spin pumping method.^[^ ^85^ ^]^ **Figure** **4**a showcases the polymer‐contained PSC device schematic illustrations. Designing route for such configuration encompass three key processes:1) injecting spins from the NiFe ferromagnetic film to the pristine semiconducting PBTTT polymer membrane by FMR‐driven spin pumping method. 2) Transmitting the excited spin angular momentum flow through the 20 nm semiconducting polymeric PBTTT layer via the inner mobile polarons. 3) Detecting the PBTTT polaron spin current flow by the ISHE‐driven electrical conversion across the large Hall angle Pt metallic electrodes. The smooth, abrupt interfaces between metals and polymer (inset of Figure 4a) offers a good premise for the injection and detection of spin information. On the other hand, large resistivity around 1.8 GΩ cm^−1^ of the 40 nm bulk PBTTT film also promises the necessary electrical isolation between the Process 1 and Process 3, hence can effectively rule out the possible electromotive contribution and short circuit influence from the NiFe terminal to the output voltage signal in Pt during ISHE measurement. For better identifying the role of the mobile polarons on carrying the spin current information, amorphous polymeric insulator CYTOP was introduced for the reference device fabrication, by stacking of NiFe/CYTOP/Pt. Resulting absent FMR signal further demonstrate the pumping‐driven spin current can only be transmitted by the polaron contributed *π*‐conjugation polymer networks instead of the unconjugated carbon–carbon linkages between the polymer repeating units. Other parallel experiment such as dependence of metallic detecting terminal spin Hall angle and external magnetic field angle were also performed, together with the PBTTT thickness influence towards V**_ISHE_** under room temperature. Resulting observations unveil that the spins can travel up to a few of hundreds nanometer in such high‐mobility, Hanle effect observable semiconducting polymer. Such diffusion value surpassed most molecule‐based spin valve measurement even until then, which owes greatly to the conductance mismatch absent feature of spin‐pumping method. Furthermore, the macroscopic *V*_ISHE_ signal as a function of temperature was afterward measured and interestingly, exhibited insensitivity to the external thermal modification. This fact set off a further discussion of polaron relaxation mechanism by reference of the diffusion‐based theoretical framework. Figuring out how spin and charge mutually influence each other in organic semiconductors during the transporting process is a long‐term puzzling also critical issue in the organic spintronics community. One direct solution is clarifying the relationship between the spin diffusion length, spin relaxation time, and charge carrier mobility under different temperatures. For the hopping‐based organic transport networks, these three key parameters can be correlated with each other uniformly by the Einstein diffusion formula, that is, *λ*_s_ = √*Dτ*_s_ where the diffusion coefficient *D* can be simulated by *D* = *µk*_B_ *T/e*. The spin diffusion length (*λ*_s_) under different temperatures, as just demonstrated, can be evaluated from the thickness‐dependent *V*_ISHE_ decay. In regard to the carrier mobility *µ*, corresponding value was obtained from the current–voltage characterization over the Ni_80_Fe_20_ (10 nm)/PBTTT (120 nm)/Pt (7 nm) tri‐layer, by combination with the space‐charge‐limited current fitting. The spin relaxation time *τ*_s_ then was extracted by the just aforementioned diffusion kinetic equation. Figure 4b showcases the contrast on temperature dependence for these key three parameters. Surprisingly, the spin diffusion length displayed temperature insensitivity in accordance with V**_ISHE_** unlike the carrier mobility which decreased with the decreasing temperature like the hopping transport. This result, in another respect, implies single enhancement on the carrier mobility of PBTTT polymer is not a necessity for improving the spin diffusion length. In addition, the spin relaxation time and the carrier mobility show the opposite temperature dependence, contradicting to the classical EY relaxation mechanism developed from the inorganic material networks.^[^ ^86^ ^]^

**Figure 5:**
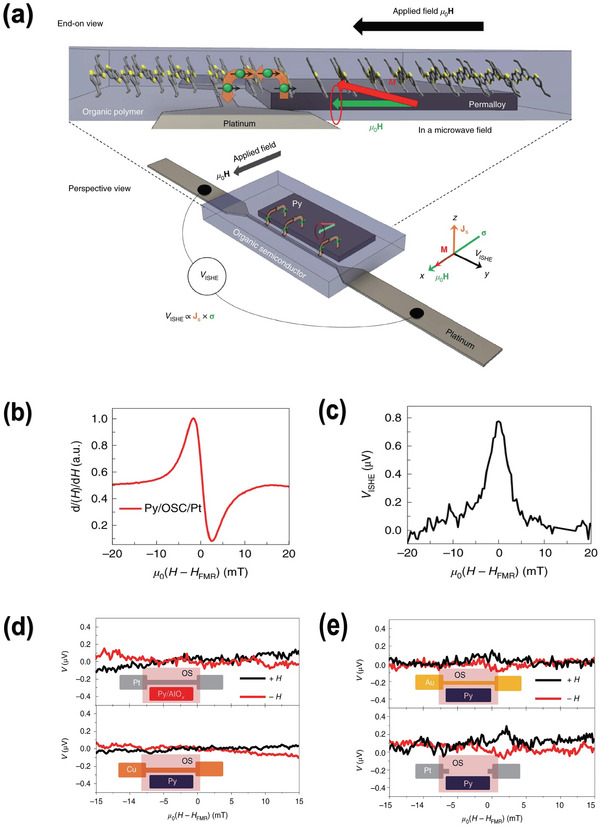
Device configuration and key signals of the lateral spin pumping device exploiting the doped polymer. a) Schematic illustration of lateral spin pumping and ISHE detection. b) External magnetic field dependence of the FMR signal measured for Py/OSC/Pt at 100 mW microwave excitation and 9.38 GHz microwave frequency inside an ESR cavity. c) Voltage response picked up by the platinum stripe when the Py island is driven into FMR. d) Field dependence of the electromotive force V measured across the metal detector for Py (25 nm)/AlOx (10 nm)/ F4TCNQ‐PBTTT/Pt (10 nm) (d, top), Py (25 nm)/F4TCNQPBTTT/Cu (10 nm) (d, bottom). e) Field dependence of the electromotive force V measured across the metal detector for Py (25 nm)/F4TCNQ‐PBTTT/Au (5 nm) (e, top) and Py (25 nm)/F4TCNQ‐PBTTT/nanofabricated broken Pt (10 nm) (e, bottom). Reproduced with permission.^[^ ^89^ ^]^ Copyright 2019, Nature Publishing Group.

**Figure 6:**
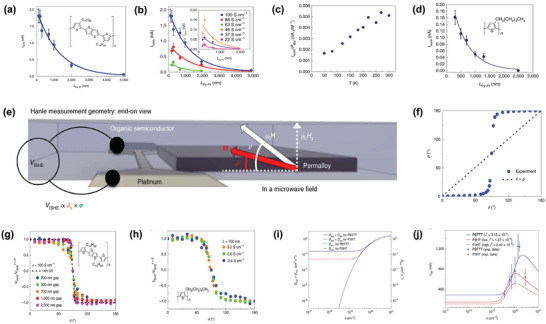
Further investigation of doping effect on the polymer based lateral spin pumping device. a) Gap distance (between Py and Pt) dependence of the ISHE‐induced current Inorm = *V*_ISHE_/*R*. b) Dependence of the measured ISHE‐induced current on the gap spacing between Py and Pt for doped F4TCNQ‐PBTTT films with different conductivities. c) Temperature dependence of the spin signal in doped PBTTT. d) Spin diffusion length extracted from lateral spin pumping in P3HT. e) Schematic geometry of Hanle measurements in lateral spin pumping devices. f) Difference in angle between the ferromagnet's magnetization and the direction of the applied field during rotation. g) Angle‐dependent measurements of *V*_ISHE_ in PBTTT for various channel lengths. h) Angle‐dependent measurements of *V*_ISHE_ in P3HT for various conductivities. i) Total diffusion coefficient, which includes contributions from hopping and exchange as a function of carrier density for PBTTT and P3HT. j) Spin diffusion length as a function of carrier density for PBTTT and P3HT. Reproduced with permission.^[^ ^89^ ^]^ Copyright 2019, Nature Publishing Group.

Deeper insight calls for the consideration of the charge trapping effect under the lower temperature. In this case, spin flips between neighboring two carrier trapping events thus the period for the trapping and detrapping process hugely impacts the relaxation time. Note that the E–Y mechanism defines the spin relaxation time decreases with the increasing temperature because of the downsized momentum scattering frequency. Such trend coincidentally concerts with the temperature dependence for the trapping events in the hopping transport organics. Naturally, trapping‐limited E–Y mechanism can give a plausible explanation to the mobility insensitive spin diffusion behavior in the discussing PBTTT polymer. Another relaxation resource namely HFI mechanism works oppositely on the temperature dependence since the enhanced local trapping sites can result in decreased *τ*_s_ under such regime. To summarize, this polaron spin current in the conjugated, undoped semiconducting polymers provide two novel viewpoints for better understanding of the spin momentum propagation in OSCs: 1) concept of improving carrier mobility leads to longer spin diffusion length is not always valid in the hopping transport dominated molecular networks. 2) Classical relaxation regimes, for instance of the E–Y mechanism, should not be used mechanically in molecule system since the mobile spin degrees are not simply transmitted or scattered by free electrons as metals. The just mentioned trapping‐limited E–Y mechanism demonstrate the importance of material characteristics, which exhibit good consistence with experimental and theoretical illustrations in the other reports.^[^ ^87^, ^88^ ^]^ However, this work still warrants further discussion given the obscurity of the hopping process assistance during the pure spin current transmission before Pt electrodes. Recently, Sirringhaus group again collaborate with other teams for further clarification of this question by aid of both spin pumping experiment and polymer doping project.^[^ ^89^ ^]^ An ultralong spin diffusion length up to 1 µm together with the fast spin transit time around 10 ns were obtained in the high spin density doped conjugated polymer. Such fascinating results were acquired from the lateral spin‐pumping device with geometry sketched in **Figure** **5**a, where the NiFe (Py) island serves as the pumping resource and the Pt wire works for ISHE electromotive detection. Such lateral pumping geometry has never been applied in the semiconducting organics before, though has been employed frequently in inorganic materials, graphene and 2D electron gas.^[^ ^90^, ^91^, ^92^ ^]^ One striking advantage compared to the classical vertical tri‐layer pumping structure, is that AHE contribution to the resulting electromotive signal can be ruled out more efficiently because of the lateral offset between spin injector and detector. Additionally, exploiting such lateral structure can preferably match the in‐plane lamellar *π*‐*π* stacking direction of the doped PBTTT polymer thin film hence theoretically speaking, promote the spin polarization retaining over longer distance. Standard pumping operation are performed in the electron spin resonance (ESR) cavity with magnetic field applied parallel to the film plane. Consequently, both the microwave absorption signal and the corresponding voltage response of Py/OSC/Pt stacking can be simultaneously measured as respectively illustrated in Figure 5b,c. Distinctive traits of FMR excitation response and V‐shape *V*_ISHE_ signal can be clearly observed thus preliminarily validate the feasibility of lateral geometry for the OSC‐based spin pumping experiment. For complete elimination of AHE contribution and potential thermal artefacts, parallel samples with insulating AlOx on coverage of Py surface was first prepared and characterized in the same ESR cavity with results shown on top of Figure 5d. Apparently, no electromotive voltage signal can be achieved once adding the AlOx layer since spin injection due to FMR was blocked. Spin Hall angle dependence of metallic detector terminal was also undertaken with alternative smallest Cu (bottom of Figure 5d) and smaller Au (bottom of Figure 5e) compared to the standard Pt detector. Observed vanishing electromotive signal also manifest the importance of decent spin Hall angle in successful lateral spin pumping operation. Finally, the coherent link between two Pt detection endings were cut off as diagrammed on the bottom of Figure 5e where no spin signal could be observed due to the absent spin current information collecting path along the conjugated polymer backbone. These comparative data powerfully supported the reliability of lateral Py/OSC/Pt structure rid of interference arising from either AHE or the other spurious electromotive origins. Based on these facts, the spin diffusion length for the F4‐TCNQ doped PBTTT was subsequently acquired by examining the spin decoherence as a function of gap distance between Py island and Pt strips (**Figure** **6**a). By fitting *I*_norm_ (*L*_Py–Pt_) = *I*_0_ e^−^ *^L^* ^Py–Pt/^ *^*λ*^* ^s^ formula, an encouraging value around 1 µm was obtained in the F4‐TCNQ doped PBTTT polymer under film conductivity of 100 S cm^−1^. Afterward, the carrier density influence on spin transport performance was comprehensively investigated to discern the responsible ingredient for long distance spin diffusion. Thermal annealing induced dopant (F4‐TCNQ) reducing^[^ ^93^ ^]^ was first attempted for disentangling the carrier density impact on the relationship between ISHE‐induced current and Py–Pt distance as summarized in Figure 6b. Undoubtedly, reduction in doped PBTTT film conductivity led to the compromising size of ISHE‐induced current similar to the influence of Py–Pt distance enlargement. However, it is too early to assert carrier density rules the spin diffusion coherence in the doped semiconducting polymer since temperature and mobility factors should also be considered for accuracy. Figure 6c showcases the temperature dependence of ISHE‐related spin signal in the doped PBTTT ranging from 50 to 300 K. As seen, decreasing temperature overall led to the decaying of current signal which can be explained by the accompanied carrier mobility downsizing. Another fact after the calculative contrast should be paid the specific attention is the entire signal attenuation extent in the temperature dependence is inferior to the situation in the just illustrated carrier density dependence. Such fact mirrors the overwhelming importance of carrier density for determining the spin diffusion length in the conjugated polymer compared to the environmental temperature and carrier mobility. Another doped polymer P3HT though possessed almost thirty times smaller conductivity than 100 S cm^−1^ doped PBTTT, only three or four hundreds nanometer smaller spin diffusion length could be obtained after extracting the data from Figure 6d. One fact should be emphasized that similar spin signals all failed to be observed in the reference undoped polymers, including both PBTTT and P3HT. Hanle measurement in such polymer contained lateral structure was also performed by tilting the angular between the film plane and the applied magnetic field. Due to the shape anisotropy within the Py film, a lagging discrepancy will occur to the magnetization orientation *Φ* compared to the manipulating out‐of‐plane angle *Θ* as clearly depicted in Figure 6e. Such difference can be read from Figure 6f where the magnetization orientation *Φ* value was obtained by aid of the Landau–Lifshitz–Gilbert equation. As depicted, in‐plane magnetization for NiFe pumping resource persists until *Θ* surpassed 60°, then quickly reorients with *Θ* before approaching 90°and finally back to in‐plane state when *Θ* tilts between 120°and 180°. According to the orthogonal principle of the inverse spin Hall effect, such uncollinear phenomenon ultimately resulted in the step‐like angular dependence as observed in both PBTTT (Figure 6g) and P3HT (Figure 6h) testing samples. Apparently, the critical points of 60°and 120°matches well with Figure 6f thus demonstrates the in‐plane spin orientation can be well maintained along the *π–π* stacking polymeric skeletons. As for the diminishing voltage signal in scope of 60°and 120°, decayed *x*‐polarized component due to enhanced Hanle precession is the main reason. These data, in another respect, unveil how these spin pumping injected spins evolves during transmission across the conjugated polymer. Back to the core issue namely the carrier density influence on the spin diffusion length, authors referred Yu's “exchange coupling” theoretical framework for disentangling how spin density influence spin diffusing activity and the resulting ISHE voltage signal.^[^ ^94^ ^]^ Under such theoretical framework, above mentioned spin diffusion coefficient *D* in formula of *λ*_s_ = √*Dτ*_s_, exactly speaking, contains both *D*_hop_ and *D*_exc_ items as *D* = *D*_hop_ + *D*_exc_ defines. *D*_hop_ denotes the spin/charge hopping and *D*_exc_ represents the exchange‐mediated coupling for spins dwelling on adjacent molecules. The fundamental difference between these two diffusion coefficient parameters is that the former *D*_hop_ requires spins moving by accompany of charge under hopping regime while the latter *D*_exc_ corresponds to the situation where spin and charge are transported separately under the premise of high carrier density. Based on this point, it can be included that spin transport in the *D*_hop_ dominant circumstance face the underlying limitation from charge carrier mobility and spin relaxation time given the Einstein relationship *D*_hop_ = *µk*_B_ *T/e*. As for the *D*_exc_ dominated case, spin information can propagate expecting longer distance by no requirement of charge motion under the prerequisite of the comparative large carrier density, which also means transfer pathways for spin angular momentum are closer and more efficient. After the theoretical modelling simulation, the Dexc was found to dominate the entire *D* by two orders of magnitude in either higher mobility doped PBTTT (*µ* ≈ 1 cm^2^ V^−1^ s^−1^) or lower mobility doped P3HT (*µ* ≈ 0.1 cm^2^ V^−1^ s^−1^) as shown in Figure 6i, under the high carrier concentration conditions. Migrating to another item in the diffusion length calculative equation *λ*_s_ = √*Dτ*_s_, the spin relaxation time *τ*_s_, whose importance on analyzing the spin diffusive behavior has been already emphasized and discussed before. Both HFI and SOC mechanisms contribute to the sum of *τ*_s_, herein, SOC regime finally works dominantly since the value for HFI field strength is no larger than 10 G based on the density functional theory in accordance with previous PBTTT research.^[^ ^83^ ^]^ Since that, spin‐mixing parameter *γ* ^2^, naturally becomes another critical influencing factor on the doped polymer's spin diffusion length which is proportional to the total SOC strength. Figure 6j displays the function between spin diffusion length and carrier density in combination with the spin‐mixing parameter based on the experimental data. As depicted, the higher carrier density affords the longer spin diffusion length in good consistence with the positive role of improved exchange coupling on enhancing spin‐preserving distance. Apart from such evidence, it can also be found that the spin diffusion length in the doped polymer is inversely proportional to the size of the spin‐mixing parameter *γ* ^2^, which can be understood at the point of the polymer backbone coplanarity. Once the doped PBTTT and P3HT has a bearing on the carrier density and *π–π* stacking distance, longer spin diffusion length will be measured for the PBTTT system since its conjugated backbone enjoying better flatness and coplanarity. Summarizing these results, clear also critical conclusion can be drawn that the spin transport phenomenon in the doped high‐mobility polymer works under the mechanism of exchange‐coupling which relies predominantly on the carrier (or spin) concentration rather than the mobile carrier mobility.

Later, Groesbeck et al. conducted a supplementary investigation on this issue in the undoped, classical also more industry‐mature OSCs yet by the spin‐pumping technique.^[^ ^95^ ^]^ Research content encompass three key techniques as follows: 1) performing routine pumping excitation and ISHE electrical conversion on FM‐polymer‐Pt trilayers to extract the spin diffusion length. 2) Measuring both the transverse and longitudinal spin relaxation time by the pulsed electrically detected magnetic resonance. 3) Charge carrier mobility for the pristine, undoped polymer was measured by the time‐of‐flight measurements. Investigating polymeric materials includes Super Yellow poly‐phenylene‐vinylene, P3HT and Polyfluorene. Meanwhile, two general organic photovoltaics fullerene acceptor molecules, namely PC_70_BM and C_70_ were also probed during spin propagation measurement. Diffusion equation was still adopted in this research while differently, diffusive coefficients for both spin (*D*_S_) and charge (*D*_c_) are probed first independently and then comparably in this work. It was found that value for *D*_S_ was significantly larger than *D*_c_ in the conjugated polymers. Situation in the small molecule fullerene acceptor is disparate where *D*_S_ and *D*_c_ values share the similar magnitude. Such difference can be partially ascribed to the higher degree of delocalization and the more interconnected transport networks nature in the conjugated OSC polymer. Huge discrepancy for the diffusion parameter of spin and charge in pristine conjugated semiconducting polymer mirrors the independent diffusive nature during the propagation. Since carrier concentration in the pristine conjugated polymer are rather low and even fail to exceed the threshold value of 10^17^ cm^3^, concept of the exchange coupling cannot be directly used for explaining the separation between charge and spin transport. Recently, Droghetti proposed another mechanism explaining the spin transport involving no charge motion in organic semiconductors, defined as the two‐fluid model which attributed such independent diffusion of charge and spin to the antiferromagnetic coupling between localized carriers’ spins.^[^ ^96^ ^]^ Such mechanism fits better to the low carrier concentration pristine polymer whose spin transport can be mediated by form of spin wave propagation somewhat like the situation in charge motion suppressed insulators.

Apart from disentangling the spin diffusive behavior in OSCs, dynamical behavior of pure spin current can even render novel insights in organic spin orbitronics, in conjunction with the chemical structure engineering project. In 2016, Sun et al. exploited the pulsed‐ISHE technique in the organic PSC research and achieved not only more significant and reliable ISHE signal, but also observed extraordinarily large SOC strength in the C_60_ molecule, which can be explained by the enhanced curvature of the molecule surface.^[^ ^97^ ^]^ Later, Liu further probed the curvature dependence of the spin diffusion length in a collection of fullerene‐based molecules, namely C_60_, C_70_, and C_84_.^[^ ^98^ ^]^ Consistent with the previous speculation by Sun, the enhanced molecular curvature in fullerene molecule led to the increased spin–orbit interaction strength and finally resulted in the decreased spin diffusion length in the spin‐pumping experiments. Differing from small molecules, torsional degree of conjugated backbone in semiconducting polymers is generally evaluated by the parameter of structural conformation or steric hindrance. Influence of polymer structural conformation on transport performance has been extensively studied in charge‐based device while rarely discussed in the spin transport issue.^[^ ^99^ ^]^ Recently, Vetter et al. performed spin pumping experiments onto three polythiophene‐based polymers in combination with the structural conformation tuning project.^[^ ^100^ ^]^ They found the decline on the polymer conjugated length can result in the simultaneous increase on the backbone torsion angles. After the comparable spin pumping experiments, they further discovered the more twisted polymer backbone is, the shorter spin diffusion length while the enhanced spin mixing conductance will be obtained. This affords a good beginning for probing the impact of polymer conjugation and conformation on the SOC strength modification together with the spin pumping‐related spin‐to‐charge conversion.

## 4 Spin Seebeck‐Induced Pure Spin Current in Organics

**Figure 7:**
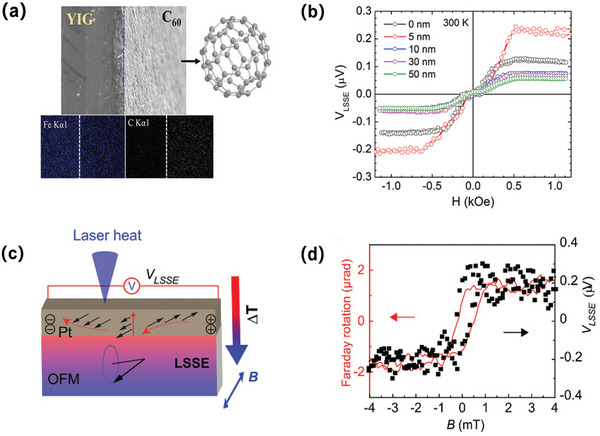
PSC induced by the spin Seebeck effect. a) Cross‐sectional SEM and EDX color map images of the 50 nm thick C_60_ deposited YIG slab. b) LSSE voltage versus magnetic field curves taken at 300 K. Reproduced with permission.^[^ ^108^ ^]^ Copyright 2020, The Royal Society. c) Schematic of the LSSE set up in ≈220 nm V(TCNE)x grown onto a 7 nm Pt layer. d) A typical ISHE response, VLSSE generated in the Pt overlayer via the LSSE in the V(TCNE)x film when sweeping an in‐plane magnetic field, which follows the magnetization switching in the V(TCNE)x film measured by a Sagnac detected magneto–optic Faraday rotation. Reproduced with permission.^[^ ^109^ ^]^ Copyright 2020, Wiley‐VCH.

Above reviewed contents all concentrate on the spin‐pumping induced pure spin current, which relied on the combination between the microwave excited magnetization and ISHE‐driven spin‐to‐charge conversion. By contrast, SSE induced thermal PSC in organics was far less reported compared to their inorganic counterpart.^[^ ^60^, ^61^, ^62^, ^63^, ^64^, ^65^, ^66^ ^]^ Let alone comparison with organic thermoelectrics field which bears a wide option on both material warehouse and processing methods.^[^ ^101^, ^102^ ^]^ Such dilemma was recently broken up by two creative reports whose explicit content will be illustrated below. YIG/Pt bilayer structure has been deeply investigated in both the FMR excitation spin‐pumping and the temperature gradient driven SSE experiments.^[^ ^103^, ^104^ ^]^ Inserting nonmagnetic spacer between the insulating, low damping YIG, and large spin Hall angel Pt films were found to have a huge influence on the interfacial spin mixing conductance also the final electromotive detection response, encompassing the thermal SSE signal.^[^ ^105^, ^106^, ^107^ ^]^ Inspired by such point, Kalappattil and colleagues tentatively placed the ball‐like C_60_ molecule between the YIG and Pt layers thus forming YIG/C_60_/Pt tri‐layer structure for herein SSE study.^[^ ^108^ ^]^ There are two main considerations for such option: the first is the large discrepancy between the spin diffusion length between 10 µm (YIG) and 2 nm (Pt), which is detrimental to the direct spin preserved transmission due to the sudden enhanced scattering. Similar sharp difference also occurs to the bulk conductance between insulating YIG and conducting Pt metal. In this case, inserting semiconducting C_60_ molecule with hundreds nanometer spin diffusion length, in principle, can alleviate the abrupt scattering enhancement for spins traveling across the YIG/Pt bilayer. **Figure** **7**a provides the cross‐sectional SEM and resulting EDX color map images to the 50‐nm C_60_ covered YIG slab. As depicted, atop C_60_ landed evenly on the surface of single crystal YIG film hence renders reliable premise to evaluate C_60_’s contribution to the entire junction's SSE signal. Room temperature LSSE data was observed as shown in Figure 7b and clearly, 5‐nm C_60_ additive spacer sample exhibited even larger *V*_LSSE_ than the YIG/Pt bilayer. Such enhancement became more prominent as the measurement temperature decreased, and also declined with the increasing C_60_ thickness which resembles the spin diffusion behavior of C_60_ itself. Enhanced LSSE after C_60_ presence can be explained by the reduced conductivity mismatch between YIG and Pt, in together with the suppressed YIG surface magnetic anisotropy supported by the supporting first principle simulations. This work knocks out the door to design molecule‐ integrated hybrid device to probe spin caloritronics or in other words, study SSE issues from organic material point of view. Nevertheless, there still exists huge shortage for the available material candidates in the organics‐based spin caloritronics. Referring this successful instance of C_60_, we can find valid OSC spacer in spin caloritronics device should encompass long spin diffusion length thus can retain thermally generated spin information over long distance before detection. Such pursuit of long spin coherence in organic media was consistent with spin transport study, yet also face the huge limit on available material option. Further interlayer engineering can be referred from the unending progress of undertaking organic spin transport items. Another protocol to bring new insights to the organic SSE phenomenon is utilizing the organic magnet as the thermal spin excitation resource.

After pumping and detecting magnons successfully and efficiently through the organic magnet, Liu subsequently undertook SSE in the low damping insulating organic magnet V(TCNE)_x_, again successfully achieved ISHE electromotive detection.^[^ ^109^ ^]^ Figure 7c gives the schematic image for such organic magnet thermally‐excited magnon spin current, where 7‐nm Pt metal was used for the LSSE signal detection neighboring to the 220‐nm V(TCNE)*_x_* bulk film under the parallel in‐plane magnetic field sweeping. Temperature gradient perpendicular to the film surface was induced by the laser heating towards single side of the V(TCNE)_x_/Pt bilayer stacking. Electrically converted signal for the resulting thermally non‐coherent spin waves was clearly illustrated in Figure 7d, whose credibility was supported by the switching behavior of V(TCNE)_x_’s magnetization (characterized by Faraday rotation angle monitoring along the film plane). Like previous organic magnon exploration, YIG/Pt classical bilayer was also prepared whose *V*_LSSE_ response still followed the magnetization hysterisis of YIG film. This gives a good indicative of the validity for the device organization and testing method during SSE investigation. Coherent microwave pumping induced organics magnons were also generated and detected, hence further demonstrate the fascinating potential of V(TCNE)*_x_*/ heavy metal structure on probing the PSC‐related issues.

Since the milestone discovery of organic giant magnetoresistance effect, organic semiconductors were endowed novel capacity on transporting spin information ranging from small molecules to recent conjugated polymers. However, such inspiring development does not repeat well in spin caloritronics study which can be explained by two main reasons. One is the lack of molecular candidates having sufficient long spin diffusion length; the other one is the absence of magnetic nature. Unlocking such dilemma requires both probing long spin coherence OSC media and developing the novel stable molecular magnet which can be processed into bulks and meets the SSE measurement demand. Recently, bi‐layer graphene was also reported to act as the spin filtering layer to control *V*_LSSE_.^[^ ^110^ ^]^ Hence we believe further progress can be achieved by aid of the cross‐links between molecular multi‐dimensional networks.

## 5 Emerging Explorations of Pure Spin Current in Organics

**Figure 8:**
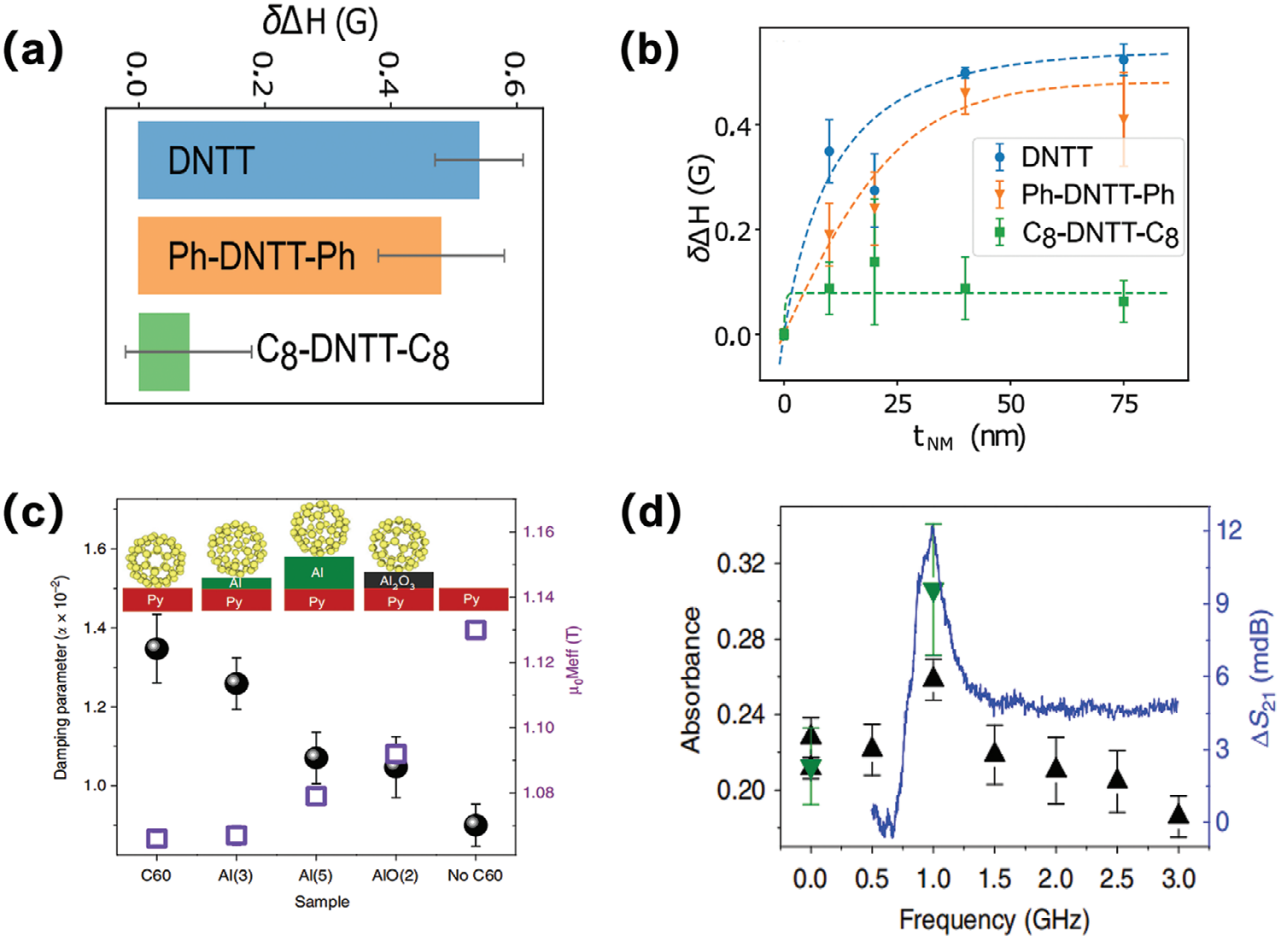
a) Bar diagram illustrating the change in linewidth for Py/DNTT (blue), Py/Ph‐DNTT‐Ph (orange), and Py/C8‐DNTTC8 (green). b) Linewidth broadening as a function of the thickness of DNTT (blue circles), Ph‐DNTT‐Ph (orange triangles), and C8‐DNTT‐C8 (green squares). From the gradual increase in signal with film thickness, the spin diffusion length can be estimated using the fit (dashed lines). Reproduced with permission.^[^ ^111^ ^]^ Copyright 2020, American Physical Society. c) Change in effective magnetization *M*_eff_ (open squares) and Gilbert damping *α* (dots) for Py with or without C_60_. d) FMR power absorption (blue line) and opticalabsorbance (triangles) versus frequency. Reproduced with permission.^[^ ^112^ ^]^ Copyright 2017, Nature Publishing Group.

The combination of the FMR‐driven spin pumping and the organic material has experienced a revolutionary progress as summarized above. Even so, unprecedented possibilities of both molecular chemical‐versatility and multifunctional or even interdisciplinary coordination remain to be explored. Undoubtedly, the spin‐pumping method works efficiently on overcoming the depolarization induced by the conductivity mismatch and the high temperature phonon scattering during the spin injection procedure under FMR excitation. Such process is generally determined by the external microwave frequency, which, on the other hand, was recently demonstrated feasible to be tuned by molecular design.^[^ ^111^ ^]^ Three dinaphtho[2,3‐b:2,3‐f]thieno[3,2‐b]thiophene(DNTT)‐based small molecule derivatives were employed for constructing the NiFe/Organic pumping‐targeted configuration in this work. Compared to the unsubstituted DNTT, C8‐DNTT‐C8, and Ph‐DNTT‐Ph contain the additive end‐substituted C8 alkyl side chains and phenyl rings separately. One critical feature for the successful spin injection at FM/OSC interface during the spin pumping experiments is the effective FMR linewidth broadening. Luckily, a significant though finite broadening on the FMR linewidth is unambiguously observed in the designed Py/DNTTs bilayer compared to the single Py bulk. This is a good premise for the further discussion since the spin angular momentum can be injected successfully also reliably from the spin pumping excited NiFe magnet to the semiconducting DNTT derivates. Explicit linewidth shift value for these three DNTT derivates are comparatively displayed in **Figure** **8**a. Authors subsequently simulated the spin mixing conductance *g* ^↑↓^ value which displayed the same chemical structure dependence as the linewidth broadening. Before clarifying the behind reason, the spin diffusion length for these three molecules was subsequently estimated via the linewidth broadening data. Resulting function between these two parameters is shown in Figure 8b, sharing the identical structure dependence as Figure 8a. The concrete value for the spin diffusion length is 40 nm (DNTT), 30 nm (Ph‐DNTT‐Ph), and 1 nm (C8‐DNTT‐C8). Thus, it can be concluded that the phenyl ring introduction though can induce a decline on the interfacial spin mixing conductance and the bulky spin diffusion length, such side effect is far less severe compared to the C8 alkyl side chain substitution. Authors later examined the molecular orientation and crystalline state of these three DNTT derivates by X‐ray based technique and AFM characterization. No distinct difference on the molecular packing motif and the aggregation states were observed, thereby triggered the following measurement on the interface energetic alignment by UPS technique for the discrimination. Interestingly, significant band bending can only be found in the C8‐DNTT except for the other two DNTT derivates. This can lead to lower spin concentration at the NiFe/organic interface and finally, narrowed the FMR linewidth broadening. Similar side effect of C8 alkyl side chain substitution on FMR response was also observed in the Py/[1] Benzothieno[3,2‐b] benzothiophene (BTBT) and Py/C8‐BTBT‐C8 reference samples. This work systematically illustrates the function and significance of molecular design on tuning spin injection efficiency under the spin pumping conditions. Vast possibilities are waiting to be unraveled for the further collaboration between the chemical structure tailoring project and the spin injection techniques.

Reviewing above PSC‐based contents, one distinct in common is the electrical conversion of injected non‐equilibrium spin current flow. Recently, Sun et al. successfully fabricated C_60_ based OSV device which even exhibiting photovoltaics performance thus unveiled the possibility of spin information processing in combination with the optical transformation and operation.^[^ ^23^ ^]^ Such exciting observation was achieved yet by aid of the electrical bias motivated spin injection and detection. Actually, charge‐free PSC generated under GHz frequency microwave excitation in the C_60_ hybrid structure was also discovered valid for the optical transformation and detection by Wheeler in 2017.^[^ ^112^ ^]^ NiFe/C_60_ was selected as the standard junction where NiFe serves as the spin pumping injection electrode under the FMR conditions. Weak hyperfine interaction, long spin diffusion length C_60_ work for the expecting optical conversion on account of its curvature enhanced SOC strength. Spin‐pumping was initially performed to check the feasibility of spin angular momentum injection and transmission. Since the pumped spin angular momentum flow was no longer electrically detected, magnetization damping increase thereby becomes the important criterion for disentangling the effective PSC injection. Figure 8c shows the change in both Gilbert damping parameter *α* (dots) and magnetization Meff (open square) dependence of NiFe/C60 interface. As seen, *α* increases by 50% when NiFe was capped onto C_60_ layer, which is an important evidence for the efficient spin current injection from NiFe to C_60_. Besides, magnitude for the pumping injected spin current intensity can be tuned by introducing spacer at the NiFe/C_60_ interface. Clearly, enhancement on *α* compared to the single NiFe is weakened when direct conductive path is hindered by the additive Al or AlOx layer. The thicker conductive Al layer was introduced, the weaker damping enhancement is obtained owing to the increased scattering events of de‐polarization. As for the sharp *α* decrease in the case of 2 nm AlOx, the absence of conductive electron for transmitting spin information is the main reason. Nevertheless, C_60_ introduction is a successful operation at least on receiving the spin information under FMR excitation. Subsequently, relationship between optical absorbance and spin pumping is probed to examine the optical conversion efficiency. In principle, the successful optical conversion for spin current demands the output optical absorbance should exceed the absorbed FMR power on magnitude. See Figure 8d, the onset successful operation started and reached the peak value at 1.0 GHz frequency, where a 30% higher light absorbance can be observed. Following decay at high frequencies can be attributed to the accompanied heating effects upon microwave irradiation. Moreover, authors also observed a THz level slowdown (0.15 THz) and a vibrational peak narrowing afterward. These findings solidly validate the feasibility of optical conversion of pumping‐induced pure spin currents and more importantly, creates another route for the organic‐based THz technique research.

## 6 Conclusion and Outlook

In summary, we summarize the past representative findings of pure spin current in the organic functional materials, including both the organics semiconductors and the organic magnets from material, conductive electron, and magnon points of view. Such exploring journey experienced a remarkable revolution from earlier borrowing lessons from the classical inorganic networks to the recent innovative combination with the spin orbitronics, molecular engineering and even optics. In spite of these impressive breakthroughs, research challenges remain in the following two main respects:

### 6.1 Material Warehouse Vacancy

Characteristics of chemical versatility for OSC material class offers unprecedented opportunities for molecule‐based electronics while such flourishing scenario made a finite influence in its spintronics counterpart. As mentioned above, valid semiconducting molecules for the spin polarized current transmission has indeed been largely extended in categories since the first reported small molecule Alq3.^[^ ^9^, ^24^ ^]^ By contrast, rather limited OSC members were applied to PSC study, despite increasing tentative reports.^[^ ^113^, ^114^, ^115^ ^]^ Behind reasons are complex which can be partially attributed to the semi‐crystalline film nature and the perplexing relationship between the transport performance and the aggregation states. The influence of charge carrier mobility on the spin angular momentum diffusive coherence has been illustrated in the above sections. Future PSC‐valid molecules exploration thus can face less limitations from charge transport respect. Furthermore, issues like molecular packing and structural conformation are likely to be more attractive during the molecule‐based spin pumping experiments. Besides, photovoltaics materials, including the soft organics and the recent hot discussing perovskite semiconductors hold great potential in the PSC‐based study owing to their extraordinary energy‐efficient background.^[^ ^116^, ^117^, ^118^, ^119^ ^]^ Moreover, we believe that the metal–organic framework (MOF) and the covalent‐organic framework (COF) materials, which have gained the massive concern and fruitful scientific findings in catalysis, energy harvesting and also optoelectronics devices,^[^ ^120^, ^121^, ^122^, ^123^ ^]^ also deserve experimental attempts since the MOF‐based organic spin valve has been successfully reported in 2020.^[^ ^124^ ^]^ Recently, spintronics nanodevices exhibiting the compatible integration with or modelling like synapses and neurons are also envisaged to mediate spin information by means of spin currents.^[^ ^3^ ^]^ Related further progress cannot be departed from the quantum computing research where lanthanide metal complexes based SMMs can serve as spin angular momentum units.^[^ ^125^, ^126^ ^]^ Likewise, biomedical spin resource for instance of organic radicals can also be anticipated to process spin current information from microscopic self‐assemble level as reported.^[^ ^127^, ^128^ ^]^

### 6.2 Suitable Organic Magnet Candidate

Over 40 years of research history, also the nontrivial part in the quantum logic device, make molecular magnet quite appealing in future spintronics and the quantum frontier realms.^[^ ^30^ ^]^ Unending endeavor from both the chemists and physicists greatly enlarge the available molecular magnet candidates for representative examples of the SMMs, the recent hot‐discussing magnetic MOF complexes and the emerging 2D organic magnets.^[^ ^129^, ^130^, ^131^, ^132^ ^]^ These emerging spin‐related materials witnessed continuous advances in not only the synthetic routes but also the optoelectronic and sensing devices together with the tunable magnetic nature and spin densities.^[^ ^31^, ^133^, ^134^, ^135^, ^136^, ^137^, ^138^ ^]^ By contrast, PSC transporting phenomenon are rarely discussed in these coordination magnetic networks despite handful theoretical predictions.^[^ ^139^, ^140^ ^]^ Such fact can be explained mainly by three reasons: 1) as for the SMM, intrinsic quantum size down to single molecule make it impossible to exploit traditional bulk heterostructures for the FMR excitation and the ISHE detection. Though successful magnetic excitation has been achieved by means of single‐molecule transistor geometry and STM technique,^[^ ^141^, ^142^, ^143^, ^144^, ^145^ ^]^ how to detect the quantum scale spin current flow effectively and precisely is another enormous challenge. Another fact cannot be ignored is the inadequate intermolecular‐level coupling of spins in quantum size SMM hence raise formidable barrier to transport spin angular momentum information coherently.^[^ ^146^, ^147^ ^]^ 2) Situation in the MOF complexes face a request of balance between the porosity and the magnetism. Conductive with high porosity MOFs can serve as either the electrode or transport channels in charge‐based functional devices, such as the supercapacitors, the chemi‐resistive sensors and the field effect transistors.^[^ ^148^ ^]^ However, the extended organic linkers rendering high porosity is somewhat detrimental to the effective exchange interaction between spin centers thus hinders successful observation of the magnetism.^[^ ^131^ ^]^ Encouragingly, both imaginary strategies and experimental solutions have been reported for successful synthesis of conducting also magnetic MOF complexes.^[^ ^132^, ^149^, ^150^, ^151^, ^152^ ^]^ Nevertheless, how to process large‐area homogeneous and impact bulk magnetic MOF membrane is the key and harsh technical problem for the stable magnet excitation as well as coherent transmission channel for spin angular momentum flow. 3) 2D magnetic inorganic materials have witnessed great advances in recent interdisciplinary research incorporating electronics, optics and spintronics.^[^ ^153^ ^]^ On the other hand, the 2D organic magnet gradually captures increasing attention due to the advantages of light‐atom components, rich flexibility in synthesis routes and potential low cost for spin information storage. Categories for the 2D organic magnet are mainly divided by two, namely metal‐contained MOF and metal‐free COF complexes. Enthusiasm for the former 2D coordination complexes centers more on the spin crossover phenomenon (transition between spin up states and spin down states) and the spin transition especially under the room temperature.^[^ ^154^, ^155^, ^156^ ^]^ By contrast, PSC through such low dimensional material networks has never been discussed partially due to the intrinsic limited crystal size of coordination complex and the poor membrane continuity as just illustrated. These characteristics together with the formidable Curie temperature engineering hinders their application as either magnetic precession motor or the effective spin transport channel. In terms of the conjugated COF complex, how to achieve detectable magnetic order experimentally is another critical issue given the spin torque generation under the metal‐free elements networks.

Regardless of these limitations, inspiring results were continuously reported thanks to the ceaseless efforts of the chemistry, physics, and material communities. Temperature‐bias driven pure spin current in SMMs was recently predicted which exhibit difference from the traditional quantum dot model.^[^ ^157^ ^]^ Another exciting discovery is the measurable ferromagnetism in 3D COF hence illustrate the possibility of embedding dynamical magnetism into the functional device.^[^ ^158^ ^]^ In addition, a strongly correlated 2D molecular magnet FeTCNQ which was synthesized by employing the superconducting precursor exhibit simultaneous capacity in magnetism, magneto‐dielectrics and photo‐irradiation induced resistance switching as well as 60 K Cuire temperature.^[^ ^159^ ^]^ To some degree, multi‐functional spintronics devices containing both the PSC transport phenomenon and the electronic or optical stimuli can be envisaged by aid of the 2D organic magnet. A foreseeable fact is the more innovative works involving PSC excited by either microwave or temperature gradient are being undertaken combined with the organic material engineering. In the long run, utilizing spin orbit torque tuning magnetization in molecular networks, designing molecule‐based MRAM chips, tuning Spin Hall magnetoresistance and even topological skyrmion‐based spin textures as well as other emergent key spin questions are expected to witness enduring miracles by probing the PSC phenomenon in organic functional materials.^[^ ^160^, ^161^, ^162^, ^163^, ^164^, ^165^ ^]^

## Conflict of Interest

The authors declare no conflict of interest.

## References

[1] S. Wolf , D. Awschalom , R. Buhrman , J. Daughton , V. S. von Molnár , M. Roukes , A. Y. Chtchelkanova , D. Treger , Science 2001, 294, 1488. 11711666 10.1126/science.1065389

[2] J. Sinova , I. Žutic , Nat. Mater. 2012, 11, 368. 22522636 10.1038/nmat3304

[3] B. Dieny , I. L. Prejbeanu , K. Garello , P. Gambardella , P. Freitas , R. Lehndorff , W. Raberg , U. Ebels , S. O. Demokritov , J. Akerman , A. Deac , P. Pirro , C. Adelmann , A. Anane , A. V. Chumak , A. Hirohata , S. Mangin , S. O. Valenzuela , M. C. Onbaşlı , M. D'Aquino , G. Prenat , G. Finocchio , L. Lopez‐Diaz , R. Chantrell , O. Chubykalo‐Fesenko , P. Bortolotti , Nat. Electron. 2020, 3, 446.

[4] J. Grollier , D. Querlioz , K. Y. Camsari , K. Everschor‐Sitte , S. Fukami , M. D. Stiles , Nat. Electron. 2020, 3, 360. 10.1038/s41928-019-0360-9 PMC7754689 33367204

[5] H. Shirakawa , A. McDiarmid , A. Heeger , Chem. Commun. 2003, 1.

[6] S. Fratini , M. Nikolka , A. Salleo , G. Schweicher , H. Sirringhaus , Nat Mater. 2020, 19, 491. 32296138 10.1038/s41563-020-0647-2

[7] K. Myny , Nat. Electron. 2018, 1, 30.

[8] V. Dediu , M. Murgia , F. C. Matacotta , C. Taliani , S. Barbanera , Solid State Commun. 2002, 122, 181.

[9] Z. H. Xiong , D. Wu , Z. V. Vardeny , J. Shi , Nature 2004, 427, 821. 14985756 10.1038/nature02325

[10] Z. V. Vardeny , Nat. Mater. 2009, 8, 91. 19165210 10.1038/nmat2366

[11] V. A. Dediu , L. E. Hueso , I. Bergenti , C. Taliani , Nat. Mater. 2009, 8, 707. 19701216 10.1038/nmat2510

[12] R. Geng , A. Roy , W. Zhao , R. C. Subedi , X. Li , J. Locklin , T. D. Nguyen , Adv. Funct. Mater. 2016, 26, 3999.

[13] H.‐J. Jang , C. A. Richter , Adv. Mater. 2017, 29, 1602739. 10.1002/adma.201602739 27859663

[14] D. Sun , E. Ehrenfreund , Z. Valy Vardeny , Chem. Commun. 2014, 50, 1781. 10.1039/c3cc47126h 24432354

[15] T. D. Nguyen , E. Ehrenfreund , Z. V. Vardeny , Science 2012, 337, 204. 22798608 10.1126/science.1223444

[16] M. Prezioso , A. Riminucci , P. Graziosi , I. Bergenti , R. Rakshit , R. Cecchini , A. Vianelli , F. Borgatti , N. Haag , M. Willis , A. J. Drew , W. P. Gillin , V. A. Dediu , Adv. Mater. 2013, 25, 534. 23097157 10.1002/adma.201202031

[17] C. Boehme , J. M. Lupton , Nat. Nanotechnol. 2013, 8, 612. 24002071 10.1038/nnano.2013.177

[18] A. Manchon , H. C. Koo , J. Nitta , S. M. Frolov , R. A. Duine , Nat. Mater. 2015, 14, 871. 26288976 10.1038/nmat4360

[19] S. Schott , E. R. McNellis , C. B. Nielsen , H.‐Y. Chen , S. Watanabe , H. Tanaka , I. McCulloch , K. Takimiya , J. Sinova , H. Sirringhaus , Nat. Commun. 2017, 8, 15200. 28492241 10.1038/ncomms15200 PMC5437270

[20] A. Soumyanarayanan , N. Reyren , A. Fert , C. Panagopoulos , Nature 2016, 539, 509. 27882972 10.1038/nature19820

[21] J. Puebla , J. Kim , K. Kondou , Y. Otani , Commun. Mater. 2020, 1, 24.

[22] Z. R. Wang , H. Q. Wu , G. W. Burr , C. S. Hwang , K. L. Wang , Q. Xia , J. J. Yang , Nat. Rev. Mater. 2020, 5, 173.

[23] X. Sun , S. Vélez , A. Atxabal , A. Bedoya‐Pinto , S. Parui , X. Zhu , R. Llopis , F. Casanova , L. E. Hueso , Science 2017, 357, 677. 28818941 10.1126/science.aan5348

[24] D. Li , G. Yu , Adv. Funct. Mater. 2021, 31, 2100550.

[25] T. P. Pareek , Phys. Rev. Lett. 2004, 92, 076601. 14995874 10.1103/PhysRevLett.92.076601

[26] T. Yang , T. Kimura , Y. Otani , Nat. Phys. 2008, 4, 851.

[27] W. Han , S. Maekawa , X. C. Xie , Nat. Mater. 2020, 19, 139. 31451780 10.1038/s41563-019-0456-7

[28] Y. Zhang , L. Guo , X. Zhu , X. Sun , Front. Chem. 2020, 8, 589207. 33195092 10.3389/fchem.2020.589207 PMC7642617

[29] A. Gaita‐Ariño , F. Luis , S. Hill , E. Coronado , Nat. Chem. 2019, 11, 301. 30903036 10.1038/s41557-019-0232-y

[30] E. Coronado , Nat. Rev. Mater. 2020, 5, 87.

[31] E. Moreno‐Pineda , W. Wernsdorfer , Nat. Rev. Phys. 2021, 3, 645.

[32] H. Li , S. Ruan , Y. J. Zeng , Adv. Mater. 2019, 31, 1900065. 10.1002/adma.201900065 31069896

[33] C. Y. Chen , J. W. Yoo , H. W. Jang , C. W. Bark , V. N. Prigodin , C. B. Eom , A. J. Epstein , Nat. Mater. 2010, 9, 638. 20639895 10.1038/nmat2797

[34] C. Cardoso , D. Soriano , N. A. García‐Martínez , J. Fernández‐ Rossier , Phys. Rev. Lett. 2018, 121, 067701. 30141640 10.1103/PhysRevLett.121.067701

[35] M. Urdampilleta , S. Klyatskaya , J. P. Cleuziou , M. Ruben , W. Wernsdorfer , Nat. Mater. 2011, 10, 502. 21685902 10.1038/nmat3050

[36] B. Huang , G. Clark , E. Navarro‐Moratalla , D. R. Klein , R. Cheng , K. L. Seyler , D. Zhong , E. Schmidgall , M. A. McGuire , D. H. Cobden , W. Yao , D. Xiao , P. Jarillo‐Herrero , X. Xu , Nature 2017, 546, 270. 28593970 10.1038/nature22391

[37] X. Chen , H. Wang , H. Liu , C. Wang , G. Wei , C. Fang , H. Wang , C. Geng , S. Liu , P. Li , H. Yu , W. Zhao , J. Miao , Y. Li , L. Wang , T. Nie , J. Zhao , X. Wu , Adv. Mater. 2022, 34, 2106172. 10.1002/adma.202106172 34816497

[38] S. Bhatti , R. Sbiaa , A. Hirohata , H. Ohno , S. Fukami , S. N. Piramanayagam , Mater. Today 2017, 20, 530.

[39] Y. Sheng , Y. Li , K. Wang , Nat. Electron. 2021, 4, 378.

[40] J. Prinzie , F. M. Simanjuntak , P. Leroux , T. Prodromakis , Nat. Electron. 2021, 4, 243.

[41] J. Lu , S. J. Poon , S. A. Wolf , B. D. Weaver , P. J. McMarr , H. Hughes , E. Chen , J. Mater. Res. 2015, 30, 1430.

[42] J. E. Hirsch , Phys. Rev. Lett. 1999, 83, 1834.

[43] Y. Kajiwara , K. Harii , S. Takahashi , J. Ohe , K. Uchida , M. Mizuguchi , H. Umezawa , H. Kawai , K. Ando , K. Takanashi , S. Maekawa , E. Saitoh , Nature 2010, 464, 262. 20220845 10.1038/nature08876

[44] A. V. Chumak , V. I. Vasyuchka , A. A. Serga , B. Hillebrands , Nat. Phys. 2015, 11, 453.

[45] P. Pirro , V. I. Vasyuchka , A. A. Serga , B. Hillebrands , Nat. Rev. Mater. 2021, 6, 1114.

[46] X. Lin , W. Yang , K. L. Wang , W. Zhao , Nat. Electron. 2019, 2, 274.

[47] G. H. Haertling , J. Am. Ceram. Soc. 1999, 82, 797.

[48] R. Cheng , J. Xiao , Q. Niu , A. Brataas , Phys. Rev. Lett. 2014, 113, 057601. 25126936 10.1103/PhysRevLett.113.057601

[49] R. H. Silsbee , A. Janossy , P. Monod , Phys. Rev. B 1979, 19, 4382.

[50] A. Azevedo , L. H. Vilela‐Leão , R. L. Rodríguez‐Suárez , A. F. LacerdaSantos , S. M. Rezende , Phys. Rev. B 2011, 83, 144402.

[51] Y. Tserkovnyak , A. Brataas , G. E. W. Bauer , Phys. Rev. B 2002, 66, 224403.

[52] B. Heinrich , C. Burrowes , E. Montoya , B. Kardasz , E. Girt , Y. Y. Song , Y. Sun , M. Wu , Phys. Rev. Lett. 2011, 107, 066604. 21902353 10.1103/PhysRevLett.107.066604

[53] L. Zhu , D. C. Ralph , R. A. Buhrman , Phys. Rev. Lett. 2019, 123, 057203. 31491309 10.1103/PhysRevLett.123.057203

[54] R. Urban , G. Woltersdorf , B. Heinrich , Phys. Rev. Lett. 2001, 87, 217204. 11736376 10.1103/PhysRevLett.87.217204

[55] S. M. Rezende , R. L. Rodríguez‐Suárez , M. M. Soares , L. H. Vilela‐Leo , D. L. Domínguez , A. Azevedo , Appl. Phys. Lett. 2013, 102, 012402.

[56] C. Hahn , G. de Loubens , M. Viret , O. Klein , V. V. Naletov , J. B. Youssef , Phys. Rev. Lett. 2013, 111, 217204. 24313523 10.1103/PhysRevLett.111.217204

[57] J. Li , C. B. Wilson , R. Cheng , M. Lohmann , M. Kavand , W. Yuan , M. Aldosary , N. Agladze , P. Wei , M. S. Sherwin , J. Shi , Nature 2020, 578, 70. 31988510 10.1038/s41586-020-1950-4

[58] X. Lv , S. Liang , L. Tao , X. Han , SPIN 2014, 4, 1440013.

[59] S. B. Riffat , X. Ma , Appl. Therm. Eng. 2003, 23, 913.

[60] T. Kikkawa , D. Reitz , H. Ito , T. Makiuchi , T. Sugimoto , K. Tsunekawa , S. Daimon , K. Oyanagi , R. Ramos , S. Takahashi , Y. Shiomi , Y. Tserkovnyak , E. Saitoh , Nat. Commun. 2021, 12, 4356. 34272371 10.1038/s41467-021-24623-6 PMC8285541

[61] C. M. Jaworski , J. Yang , S. Mack , D. D. Awschalom , J. P. Heremans , R. C. Myers , Nat. Mater. 2010, 9, 898. 20871608 10.1038/nmat2860

[62] K. Uchida , J. Xiao , H. Adachi , J. Ohe , S. Takahashi , J. Ieda , T. Ota , Y. Kajiwara , H. Umezawa , H. Kawai , G. E. Bauer , S. Maekawa , E. Saitoh , Nat. Mater. 2010, 9, 894. 20871606 10.1038/nmat2856

[63] G. E. Bauer , E. Saitoh , B. J. van Wees , Nat. Mater. 2012, 11, 391. 22522639 10.1038/nmat3301

[64] A. Hoffmann , S. D. Bader , Phys. Rev. Appl. 2015, 4, 047001.

[65] D. Meier , D. Reinhardt , M. Van Straaten , C. Klewe , M. Althammer , M. Schreier , S. T. B. Goennenwein , A. Gupta , M. Schmid , C. H. Back , Nat. Commun. 2015, 6, 8211. 26394541 10.1038/ncomms9211 PMC4598359

[66] K. Uchida , M. Ishida , T. Kikkawa , A. Kirihara , T. Murakami , E. Saitoh , J Phys Condens Matter 2014, 26, 343202. 25105889 10.1088/0953-8984/26/34/343202

[67] D. D. Awschalom , M. E. Flatte , Nat. Phys. 2007, 3, 153.

[68] T. Jungwirth , J. Wunderlich , V. Novák , K. Olejník , B. L. Gallagher , R. P. Campion , K. W. Edmonds , A. W. Rushforth , A. J. Ferguson , P. Němec , Rev. Mod. Phys. 2014, 86, 855.

[69] K. Ando , S. Watanabe , S. Mooser , E. Saitoh , H. Sirringhaus , Nat. Mater. 2013, 12, 622. 23644525 10.1038/nmat3634

[70] K. Ando , E. Saitoh , Phys. Rev. Lett. 2012, 109, 026602. 23030190 10.1103/PhysRevLett.109.026602

[71] S. Jiang , S. Liu , P. Wang , Z. Luan , X. Tao , H. Ding , D. Wu , Phys. Rev. Lett. 2015, 115, 086601. 26340196 10.1103/PhysRevLett.115.086601

[72] Y. Tani , Y. Teki , E. Shikoh , Appl. Phys. Lett. 2015, 107, 242406.

[73] Y. Tanaka , T. Kono , Y. Teki , E. Shikoh , IEEE Trans. Magnetics 2019, 55, 18379261.

[74] H. Liu , J. Wang , M. Groesbeck , X. Pan , C. Zhang , Z. V. Vardeny , J. Mater. Chem. C 2018, 6, 3621.

[75] J. B. S. Mendes , O. Alves Santos , J. P. Gomes , H. S. Assis , J. F. Felix , R. L. Rodríguez‐Suárez , S. M. Rezende , A. Azevedo , Phys. Rev. B 2017, 95, 014413.

[76] G. Schmidt , C. Hauser , P. Trempler , M. Paleschke , E. T. Papaioannou , Phys. Status Solidi B 2020, 257, 1900644.

[77] J. S. Miller , Chem. Soc. Rev. 2011, 40, 3266.21479292

[78] J. W. Yoo , C. Y. Chen , H. W. Jang , C. W. Bark , V. N. Prigodin , C. B. Eom , A. J. Epstein , Nat. Mater. 2010, 9, 638. 20639895 10.1038/nmat2797

[79] B. Li , C.‐Y. Kao , J.‐W. Yoo , V. N. Prigodin , A. J. Epstein , Adv. Mater. 2011, 23, 3382. 21721052 10.1002/adma.201100903

[80] B. Li , J.‐W. Yoo , C.‐Y. Kao , H. W. Jang , C.‐B. Eom , A. J. Epstein , Org. Electron. 2010, 11, 1149.

[81] H. Liu , C. Zhang , H. Malissa , M. Groesbeck , M. Kavand , R. McLaughlin , S. Jamali , J. Hao , D. Sun , R. A. Davidson , L. Wojcik , J. S. Miller , C. Boehme , Z. V. Vardeny , Nat. Mater. 2018, 17, 308. 29531369 10.1038/s41563-018-0035-3

[82] Z. G. Yu , Phys. Rev. Lett. 2011, 106, 106602. 21469820 10.1103/PhysRevLett.106.106602

[83] Z. G. Yu , F. Ding , H. Wang , Phys. Rev. B 2013, 87, 205446.

[84] S. Ding , Y. Tian , W. Hu , Nano Res. 2021, 14, 3653.

[85] S. Watanabe , K. Ando , K. Kang , S. Mooser , Y. Vaynzof , H. Kurebayashi , E. Saitoh , H. Sirringhaus , Nat. Phys. 2014, 10, 308.

[86] S. Pramanik , C.‐G. Stefanita , S. Patibandla , S. Bandyopadhyay , K. Garre , N. Harth , M. Cahay , Nat. Nanotechnol. 2007, 2, 216. 18654265 10.1038/nnano.2007.64

[87] Z. G. Yu , Nat. Commun. 2014, 5, 4842. 25203690 10.1038/ncomms5842

[88] H. Matsui , D. Kumaki , E. Takahashi , K. Takimiya , S. Tokito , T. Hasegawa , Phys. Rev. B 2012, 85, 035308.

[89] S.‐J. Wang , D. Venkateshvaran , M. R. Mahani , U. Chopra , E. R. McNellis , R. D. Pietro , S. Schott , A. Wittmann , G. Schweicher , M. Cubukcu , K. Kang , R. Carey , T. J. Wagner , J. N. M. Siebrecht , D. P. G. H. Wong , I. E. Jacobs , R. O. Aboljadayel , A. Ionescu , S. A. Egorov , S. Mueller , O. Zadvorna , P. Skalski , C. Jellett , M. Little , A. Marks , I. McCulloch , J. Wunderlich , J. Sinova , H. Sirringhaus , Nat. Electron. 2019, 2, 98.

[90] A. Yamamoto , Y. Ando , T. Shinjo , T. Uemura , M. Shiraishi , Phys. Rev. B 2015, 91, 024417.

[91] Z. Tang , E. Shikoh , H. Ago , K. Kawahara , Y. Ando , T. Shinjo , M. Shiraishi , Phys. Rev. B 2013, 87, 140401.

[92] R. Ohshima , Y. Ando , K. Matsuzaki , T. Susaki , M. Weiler , S. Klingler , H. Huebl , E. Shikoh , T. Shinjo , S. T. B. Goennenwein , M. Shiraishi , Nat. Mater. 2017, 16, 609. 28191896 10.1038/nmat4857

[93] K. Kang , S. Watanabe , K. Broch , A. Sepe , A. Brown , I. Nasrallah , M. Nikolka , Z. Fei , M. Heeney , D. Matsumoto , K. Marumoto , H. Tanaka , S.‐I. Kuroda , H. Sirringhaus , Nat. Mater. 2016, 15, 896. 27159015 10.1038/nmat4634

[94] Z. G. Yu , Phys. Rev. Lett. 2013, 111, 016601. 23863018 10.1103/PhysRevLett.111.016601

[95] M. Groesbeck , H. Liu , M. Kavand , E. Lafalce , J. Wang , X. Pan , T. H. Tennahewa , H. Popli , H. Malissa , C. Boehme , Z. V. Vardeny , Phys. Rev. Lett. 2020, 124, 067702. 32109121 10.1103/PhysRevLett.124.067702

[96] A. Droghetti , S. Sanvito , Phys. Rev. B 2019, 99, 094413.

[97] D. Sun , K. J. van Schooten , M. Kavand , H. Malissa , C. Zhang , M. Groesbeck , C. Boehme , Z. V. Vardeny , Nat. Mater. 2016, 15, 863. 27088233 10.1038/nmat4618

[98] H. Liu , J. Wang , A. Chanana , Z. V. Vardeny , J. Appl. Phys. 2019, 125, 142908.

[99] J. Y. Huang , G. Yu , Chem. Mater. 2021, 33, 1513.

[100] E. Vetter , I. VonWald , S. Yang , L. Yan , S. Koohfar , D. Kumah , Z. G. Yu , W. You , D. Sun , Phys. Rev. Mater. 2020, 4, 085603.

[101] W. Jin , L. Liu , T. Yang , H. Shen , J. Zhu , W. Xu , S. Li , Q. Li , L. Chi , C.‐A. Di , D. Zhu , Nat. Commun. 2018, 9, 3586. 30181592 10.1038/s41467-018-05999-4 PMC6123419

[102] Y. Zhao , L. Liu , F. Zhang , C.‐A. Di , D. Zhu , SmartMat 2021, 2, 426.

[103] L. Liu , Y. Li , Y. Liu , T. Feng , J. Xu , X. R. Wang , D. Wu , P. Gao , J. Li , Phys. Rev. B 2020, 102, 014411.

[104] A. Sola , V. Basso , M. Kuepferling , C. Dubs , M. Pasquale , Sci. Rep. 2019, 9, 2047. 30765855 10.1038/s41598-019-38687-4 PMC6376020

[105] L. Frangou , S. Oyarzún , S. Auffret , L. Vila , S. Gambarelli , V. Baltz , Phys. Rev. Lett. 2016, 116, 077203. 26943556 10.1103/PhysRevLett.116.077203

[106] J. Cramer , F. Fuhrmann , U. Ritzmann , V. Gall , T. Niizeki , R. Ramos , Z. Qiu , D. Hou , T. Kikkawa , J. Sinova , U. Nowak , E. Saitoh , M. Kläui , Nat. Commun. 2018, 9, 1089. 29540718 10.1038/s41467-018-03485-5 PMC5852167

[107] D. Kikuchi , M. Ishida , K. Uchida , Z. Qiu , T. Murakami , E. Saitoh , Appl. Phys. Lett. 2015, 106, 082401.

[108] V. Kalappattil , R. Geng , R. Das , M. Pham , H. Luong , T. Nguyen , A. Popescu , L. M. Woods , M. Kläui , H. Srikanth , M. H. Phan , Mater. Horiz. 2020, 7, 1413.

[109] H. Liu , H. Malissa , R. M. Stolley , J. Singh , M. Groesbeck , H. Popli , M. Kavand , S. K. Chong , V. V. Deshpande , J. S. Miller , C. Boehme , Z. V. Vardeny , Adv. Mater. 2020, 32, 2002663. 10.1002/adma.202002663 32844503

[110] W. Y. Lee , M. S. Kang , G. S. Kim , N. W. Park , K. Y. Choi , C. T. Le , M. U. Rashid , E. Saitoh , Y. S. Kim , S. K. Lee , ACS Appl. Mater. Interfaces 2021, 13, 45097. 34496563 10.1021/acsami.1c13180

[111] A. Wittmann , G. Schweicher , K. Broch , J. Novak , V. Lami , D. Cornil , E. R. McNellis , O. Zadvorna , D. Venkateshvaran , K. Takimiya , Y. H. Geerts , J. Cornil , Y. Vaynzof , J. Sinova , S. Watanabe , H. Sirringhaus , Phys. Rev. Lett. 2020, 124, 027204. 32004034 10.1103/PhysRevLett.124.027204

[112] M. C. Wheeler , F. Al Ma'Mari , M. Rogers , F. J. Gonçalves , T. Moorsom , A. Brataas , R. Stamps , M. Ali , G. Burnell , B. J. Hickey , O. Cespedes , Nat. Commun. 2017, 8, 926. 29030558 10.1038/s41467-017-01034-0 PMC5640639

[113] H. Liu , M. Groesbeck , E. Lafalce , X. Liu , Z. V. Vardeny , J. Photonics Energy 2018, 8, 032212.

[114] T. Li , L. Xu , X. Xiao , F. Chen , L. Cao , W. Wu , W. Tong , F. Zhang , ACS Appl. Mater. Interfaces 2020, 12, 2708. 31894693 10.1021/acsami.9b16602

[115] T. Manoj , S. Kotha , B. Paikaray , D. Srideep , A. Haldar , K. V. Rao , C. Murapaka , RSC Adv. 2021, 11, 35567. 35493144 10.1039/d1ra07349d PMC9043263

[116] L. Zhu , M. Zhang , J. Xu , C. Li , J. Yan , G. Zhou , W. Zhong , T. Hao , J. Song , X. Xue , Z. Zhou , R. Zeng , H. Zhu , C.‐C. Chen , R. C. I. MacKenzie , Y. Zou , J. Nelson , Y. Zhang , Y. Sun , F. Liu , Nat. Mater. 2022, 21, 656. 35513501 10.1038/s41563-022-01244-y

[117] Y. Liu , B. Liu , C.‐Q. Ma , F. Huang , G. Feng , H. Chen , J. Hou , L. Yan , Q. Wei , Q. Luo , Q. Bao , W. Ma , W. Liu , W. Li , X. Wan , X. Hu , Y. Han , Y. Li , Y. Zhou , Y. Zou , Y. Chen , Y. Li , Y. Chen , Z. Tang , Z. Hu , Z.‐G. Zhang , Z. Bo , Sci. China: Chem. 2022, 65, 224.

[118] J. Jeong , M. Kim , J. Seo , H. Lu , P. Ahlawat , A. Mishra , Y. Yang , M. A. Hope , F. T. Eickemeyer , M. Kim , Y. J. Yoon , I. W. Choi , B. P. Darwich , S. J. Choi , Y. Jo , J. H. Lee , B. Walker , S. M. Zakeeruddin , L. Emsley , U. Rothlisberger , A. Hagfeldt , D. S. Kim , M. Grätzel , J. Y. Kim , Nature 2021, 592, 381. 33820983 10.1038/s41586-021-03406-5

[119] Y. Zhou , L. M. Herz , A. K. Y. Jen , M. Saliba , Nat. Energy 2022, 7, 794.

[120] L. Wang , S. E. Saji , L. Wu , Z. Wang , Z. Chen , Y. Du , X. Yu , H. Zhao , Z. Yin , Small 2022, 18, 2201642. 10.1002/smll.202201642 35843870

[121] C. Li , D. Li , W. Zhang , H. Li , G. Yu , Angew. Chem., Int. Ed. 2021, 60, 27135. 10.1002/anie.202112924 34585820

[122] R. Freund , O. Zaremba , G. Arnauts , R. Ameloot , G. Skorupskii , M. Dincă , A. Bavykina , J. Gascon , A. Ejsmont , J. Goscianska , M. Kalmutzki , U. Lächelt , E. Ploetz , C. S. Diercks , S. Wuttke , Angew. Chem., Int. Ed. 2021, 60, 23975. 10.1002/anie.202106259 33989445

[123] X. F. Lu , Y. Fang , D. Luan , X. W. D. Lou , Nano Lett. 2021, 21, 1555. 33567819 10.1021/acs.nanolett.0c04898

[124] X. Song , X. Wang , Y. Li , C. Zheng , B. Zhang , C.‐A. Di , F. Li , C. Jin , W. Mi , L. Chen , W. Hu , Angew. Chem., Int. Ed. 2020, 59, 1118. 10.1002/anie.201911543 31659842

[125] C. Godfrin , A. Ferhat , R. Ballou , S. Klyatskaya , M. Ruben , W. Wernsdorfer , F. Balestro , Phys. Rev. Lett. 2017, 119, 187702. 29219608 10.1103/PhysRevLett.119.187702

[126] C. Gao , A. Genoni , S. Gao , S. Jiang , A. Soncini , J. Overgaard , Nat. Chem. 2020, 12, 213. 31844195 10.1038/s41557-019-0387-6

[127] M. Mas‐Torrent , N. Crivillers , V. Mugnaini , I. Ratera , C. Rovira J Veciana , J. Mater. Chem. 2009, 19, 1691.

[128] D.‐H. Tuo , S. Tang , P. Jin , J. Li , X. Wang , C. Zhang , Y.‐F. Ao , Q.‐Q. Wang , D.‐X. Wang , CCS Chem. 2022, 10.31635/ccschem.022.202202167.

[129] L. Bogani , W. Wernsdorfer , Nat. Mater. 2008, 7, 179. 18297126 10.1038/nmat2133

[130] G. Molnar , S. Rat , L. Salmon , W. Nicolazzi , A. Bousseksou , Adv. Mater. 2018, 30, 1703862. 10.1002/adma.201703862 29171924

[131] A. E. Thorarinsdottir , T. D. Harris , Chem. Rev. 2020, 120, 8716. 32045215 10.1021/acs.chemrev.9b00666

[132] J. Xing , P. Wang , Z. Jiang , X. Jiang , Y. Wang , J. Zhao , APL Mater. 2020, 8, 071105.

[133] F.‐S. Guo , B. M. Day , Y.‐C. Chen , M.‐L. Tong , A. Mansikkamäki , R. A. Layfield , Science 2018, 362, 1400. 30337456 10.1126/science.aav0652

[134] M. Gavara‐Edo , R. Córdoba , F. J. Valverde‐Muñoz , J. Herrero‐Martín , J. A. Real , E. Coronado , Adv. Mater. 2022, 34, 2202551. 10.1002/adma.202202551 35766419

[135] W. Liu , Y.‐Y. Peng , S.‐G. Wu , Y.‐C. Chen , M. N. Hoque , Z.‐P. Ni , X.‐M. Chen , M.‐L. Tong , Angew. Chem., Int. Ed. 2017, 56, 14982. 10.1002/anie.201708973 28967999

[136] J. Zhang , W. Kosaka , Y. Kitagawa , H. Miyasaka , Nat. Chem. 2021, 13, 191. 33257884 10.1038/s41557-020-00577-y

[137] G. M. Espallargas , E. Coronado , Chem. Soc. Rev. 2018, 47, 533. 29112210 10.1039/c7cs00653e

[138] K. Senthil Kumar , M. Ruben , Coord. Chem. Rev. 2017, 346, 176.

[139] B. Luo , J. Liu , J.‐T. Lü , J.‐H. Gao , K.‐L. Yao , Sci. Rep. 2014, 4, 4128. 24549224 10.1038/srep04128 PMC3928577

[140] P.‐B. Niu , L.‐X. Liu , Z. Sun , X.‐Q. Su , L.‐J. Dong , H.‐G. Luo , J. Magn. Magn. Mater. 2018, 465, 9.

[141] H. B. Heersche , Z. de Groot , J. A. Folk , H. S. J. van der Zant , C. Romeike , M. R. Wegewijs , L. Zobbi , D. Barreca , E. Tondello , A. Cornia , Phys. Rev. Lett. 2006, 96, 206801. 16803192 10.1103/PhysRevLett.96.206801

[142] A. S. Zyazin , J. W. G. van den Berg , E. A. Osorio , H. S. J. van der Zant , N. P. Konstantinidis , M. Leijnse , M. R. Wegewijs , F. May , W. Hofstetter , C. Danieli , A. Cornia , Nano Lett. 2010, 10, 3307. 20687519 10.1021/nl1009603

[143] R. Gaudenzi , E. Burzurí , D. Reta , I. D. P. Moreira , S. T. Bromley , C. Rovira , J. Veciana , H. S. Van Der Zant , Nano Lett. 2016, 16, 2066. 26862681 10.1021/acs.nanolett.6b00102

[144] B. M. Heinrich , L. Braun , J. I. Pascual , K. J. Franke , Nano Lett. 2015, 15, 4024. 25942560 10.1021/acs.nanolett.5b00987

[145] B. M. Heinrich , L. Braun , J. I. Pascual , K. J. Franke , Nat. Phys. 2013, 9, 765.

[146] Y.‐C. Chen , M.‐L. Tong , Chem. Sci. 2022, 13, 8716. 35975153 10.1039/d2sc01532c PMC9350631

[147] P. Willke , T. Bilgeri , X. Zhang , Y. Wang , C. Wolf , H. Aubin , A. Heinrich , T. Choi , ACS Nano 2021, 15, 17959. 34767351 10.1021/acsnano.1c06394

[148] W.‐H. Li , W.‐H. Deng , G.‐E. Wang , G. Xu , EnergyChem 2020, 2, 100029.

[149] X. Li , J. Yang , J. Am. Chem. Soc. 2019, 141, 109. 30580516 10.1021/jacs.8b11346

[150] H. Lv , X. Li , D. Wu , Y. Liu , X. Li , X. Wu , J. Yang , Nano Lett. 2022, 22, 1573. 35148110 10.1021/acs.nanolett.1c04398

[151] R. Dong , Z. Zhang , D. C. Tranca , S. Zhou , M. Wang , P. Adler , Z. Liao , F. Liu , Y. Sun , W. Shi , Z. Zhang , E. Zschech , S. C. B. Mannsfeld , C. Felser , X. Feng , Nat. Commun. 2018, 9, 2637. 29980687 10.1038/s41467-018-05141-4 PMC6035257

[152] K. Siemensmeyer , C. A. Peeples , P. Tholen , F.‐J. Schmitt , B. Çoşut , G. Hanna , G. Yücesan , Adv. Mater. 2020, 32, 2000474. 10.1002/adma.202000474 32374449

[153] Q. H. Wang , A. Bedoya‐Pinto , M. Blei , A. H. Dismukes , A. Hamo , S. Jenkins , M. Koperski , Y. Liu , Q. Sun , E. J. Telford , H. H. Kim , M. Augustin , U. Vool , J. X. Yin , L. H. Li , A. Falin , C. R. Dean , F. Casanova , R. F. L. Evans , M. Chshiev , A. Mishchenko , C. Petrovic , R. He , L. Zhao , A. W. Tsen , B. D. Gerardot , M. Brotons‐Gisbert , Z. Guguchia , X. Roy , S. Tongay , et al., ACS Nano 2022, 16, 6960. 35442017 10.1021/acsnano.1c09150 PMC9134533

[154] F. Prins , M. Monrabal‐Capilla , E. A. Osorio , E. Coronado , H. S. J. Van Der Zant , Adv. Mater. 2011, 23, 1545. 21449059 10.1002/adma.201003821

[155] E. Coronado , M. Giménez‐Marqués , G. Mínguez Espallargas , F. Rey , I. J. Vitórica‐Yrezábal , J. Am. Chem. Soc. 2013, 135, 15986. 24125096 10.1021/ja407135k

[156] A. Holovchenko , J. Dugay , M. Giménez‐Marqués , R. Torres‐ Cavanillas , E. Coronado , H. S. J. van der Zant , Adv. Mater. 2016, 28, 7228. 27184546 10.1002/adma.201600890

[157] P. Niu , L. Liu , X. Su , L. Dong , H.‐G. Luo , AIP Adv. 2018, 8, 015215.

[158] S. Wang , X.‐X. Li , L. Da , Y. Wang , Z. Xiang , W. Wang , Y.‐B. Zhang , D. Cao , J. Am. Chem. Soc. 2021, 143, 15562. 34533316 10.1021/jacs.1c06986

[159] Y. Huang , S. Zhang , G. Zhong , Y. Hu , H. Zhou , F. Hu , C. Li , R. Yang , Z. Li , J. N. Armstrong , S. Ren , Matter 2020, 2, 1639.

[160] H. Wu , A. Chen , P. Zhang , H. He , J. Nance , C. Guo , J. Sasaki , T. Shirokura , P. N. Hai , B. Fang , S. A. Razavi , K. Wong , Y. Wen , Y. Ma , G. Yu , G. P. Carman , X. Han , X. Zhang , K. L. Wang , Nat. Commun. 2021, 12, 6251. 34716324 10.1038/s41467-021-26478-3 PMC8556271

[161] D. Sun , Y. Zhai , K. J. van Schooten , C. Zhang , M. Kavand , H. Malissa , M. Groesbeck , R. Menon , C. Boehme , Z. V. Vardeny , J. Phys.: Condens. Matter 2018, 30, 484003. 30418948 10.1088/1361-648X/aae86f

[162] D. L. Sun , C. M. Kareis , K. J. van Schooten , W. Jiang , G. Siegel , M. Kavand , R. A. Davidson , W. W. Shum , C. Zhang , H. L. Liu , A. Tiwari , C. Boehme , F. Liu , P. W. Stephens , J. S. Miller , Z. V. Vardeny , Phys. Rev. B 2017, 95, 054423.

[163] Y. Tikhonov , S. Kondovych , J. Mangeri , M. Pavlenko , L. Baudry , A. Sene , A. Galda , S. Nakhmanson , O. Heinonen , A. Razumnaya , I. Luk'yanchuk , V. M. Vinokur , Sci. Rep. 2020, 10, 8657. 32457537 10.1038/s41598-020-65291-8 PMC7251125

[164] J. Hellsvik , R. D. Perez , R. M. Geilhufe , M. Månsson , A. V. Balatsky , Phys. Rev. Mater. 2020, 4, 024409.

[165] H. Lian , X. Cheng , H. Hao , J. Han , M.‐T. Lau , Z. Li , Z. Zhou , Q. Dong , W.‐Y. Wong , Chem. Soc. Rev. 2022, 51, 1926. 35083990 10.1039/d0cs00569j

